# Targeting histone deacetylation, cell cycle regulators and heat shock proteins as novel therapeutic strategies for penile cancers

**DOI:** 10.1038/s41698-026-01391-4

**Published:** 2026-03-31

**Authors:** Lara Marson, Margaretha A. Skowron, Pailin Pongratanakul, Mara Kotthoff, Gereon Poschmann, Hanibal Bohnenberger, Alexa Stephan, Meike M. Watolla, Elvira Mukinovic, Kai Stühler, Thomas Kurz, Hiresh Ayoubian, Johannes Linxweiler, Kerstin Junker, Daniel Nettersheim

**Affiliations:** 1 Department of Urology, Urological Research Laboratory, Translational UroOncology, Medical Faculty and University Hospital Düsseldorf, Heinrich Heine University Düsseldorf, Düsseldorf, Germany; 2 Molecular Proteomics Laboratory (MPL), Biological and Medical Research Center (BMFZ), Medical Faculty and University Hospital Düsseldorf, Heinrich Heine University, Düsseldorf, Germany; 3 Institute of Pathology, University Medical Center Göttingen, Göttingen, Germany; 4 Department of Pharmaceutical and Medical Chemistry, Heinrich Heine University Düsseldorf, Düsseldorf, Germany; 5 Department of Urology and Pediatric Urology, Saarland University, Homburg, Germany; 6 Center for Integrated Oncology Aachen Bonn Cologne Düsseldorf (CIO ABCD), Düsseldorf, Germany

**Keywords:** Cancer, Drug discovery, Oncology

## Abstract

Penile carcinoma (PeCa) is a rare malignancy occurring in men. Treatment typically involves penectomy, radiation, or cisplatin-based chemotherapy. A paclitaxel, ifosfamide, and cisplatin-based regimen achieves response rates around 65%. However, non-responders face a 5-year survival rate of only 8%, and due to its rarity, it is mediocrely characterized. Thus, an in-depth delineation of PeCa is crucial to identify novel therapeutic targets. Newly established primary human PeCa cell lines and corresponding xenografts tumors were examined using mass spectrometry and phospho-kinase arrays to analyze the (phospho-)proteome and secretome. Proteomic analyses identified heat shock proteins (HSP27/60/70), factors involved in posttranslational modifications (e.g. acetylation), and the VEGF signaling pathway as putative therapeutic targets. Secreted factors were associated with the HIF-1 and Hippo signaling cascades. Compared to standard chemotherapy (e.g. cisplatin, 5-FU, ifosfamide, irinotecan), treatment with romidepsin, quisinostat (HDAC inhibitors (i)), palbociclib (CDK4/6i), or 17-AAG and PU-H71 (HSP90i) reduced cell viability, induced apoptosis, and led to G2 / M cell cycle arrest in most PeCa cells. This research underscores the therapeutic potential of using HDAC, CDK4/6, and HSP90 inhibitors for PeCa management and reveals additional promising targets and biomarkers for future strategies.

## Introduction

Representing a rare malignant disease, penile cancer (PeCa) represents 0.4 - 0.6% of male neoplasias in Europe and the USA^1,2^. Though PeCa accounts for up to 10% of neoplasias in developing regions, such as African and South American countries or India, eventually resulting in about 36,000 cases worldwide^1,2^. With more than 95%, most PeCa originate from squamous cells of the glans and preputial skin. These squamous cell carcinomas (SCC) can be further distinguished based on whether they are human papillomavirus (HPV)-driven or not. While non-HPV-related PeCa can be classified into the usual type, pseudohyperplastic, pseudoglandular, verrucous, papillary, adenosquamous, or sarcomatoid squamous carcinomas, HPV^+^ PeCa often present with basaloid-, papillary / basaloid-, warty / basaloid-, clear cell-, or lymphoepithelioma-like carcinoma^3–7^. PeCa incidences were shown to correlate with low hygiene and educational standards. Even though age represents another risk factor to develop PeCa with a peaking incidence in the >60 year age group^1^, no statistical significance was observed regarding the disease-specific-, recurrence-free, or metastasis-free survival in male PeCa patients aged either below or above 50 years^8^. Additionally, phimosis, smoking, balantis xerotica obliterans, penile trauma, and lack of neonatal circumcision are known risk factors to develop PeCa^1,2,7^. Moreover, a recent Norwegian study highlighted the association between a high body mass index and an increased risk of PeCa development^9^. However, sexually transmitted HPV represents with 15–80% of PeCa patients the most common risk factor with increasing numbers of HPV^+^ PeCa over the past 50 years^10^. While HPV 16 and 18 are the most common HPV-subtypes found in PeCa, HPV 6, 11, 16, 18, 31, 33, and 35 have been documented in penile intraepithelial neoplasia^5^.

Upon infection of the penile epithelial mucosal cells, the HPV integrates its DNA into the host genome, eventually resulting in the translation of the viral oncogenes E6 and E7, which inactivate the tumor suppressors Rb1 and p53. Moreover, the chronic inflammatory response to the HPV infection further results in genomic instability, therefore, promoting a malignant transformation of the cell^4^. Non-HPV-related PeCa have been ascribed to arise from chronic inflammation via COX2-signaling, eventually resulting in the development of DNA damage- and genomic instability-inducing reactive oxygen species^4,5^.

The most frequent single-nucleotide variants (SNV) found in PeCa samples were C > T transitions, which resembled the COSMIC (Catalogue of Somatic Mutations in Cancer) signatures SBS2 (APOBEC cytidine deaminase activity) or SBS6 (defective DNA mismatch repair). Moreover, this APOBEC-related mutational pattern correlated with a higher tumor mutational burden (TMB) and worse overall survival (OS) as compared to the non-APOBEC-enriched profiles^11^. Also, Zhou et al. observed in their whole-genome sequencing of newly established PeCa cell lines most frequently transitions in C > T and T > C^12^. Several studies have investigated putative driver mutations or aberrant signaling cascades promoting PeCa development. Here, *CDKN2A, CDKN2B, FAT1, GSTM1, GSTP1, HRAS, KRAS, MYCN, NOTCH1, PIK3CA, TERT,* and *TP53* were found to be among the most mutated genes in PeCa samples^5,6,11–25^. As such, *CCND1*, *FAK*, or *MYCN* amplifications correlated with a worse prognosis of PeCa patients^22,26^. *HRAS*, *KRAS*, and *PIK3CA* mutations were observed to be crucial during the development of *TP53*^-^ PeCa^23^. Further genomic alterations in metastatic PeCa included aberrant mTOR signaling, DNA repair, as well as signaling cascades involving EGFR, ERBB2/HER2, and FGFR3^27^. Generally, the TMB was rather low in PeCa (mean 1.96 SNV/Mb, ranging 0.01 – 21.2 SNV / Mb) and was comparable to the TMB of cervical, esophageal, and head and neck squamous cell carcinoma^11^. Though a high TMB (> 10 mutations / Mb) was observed in metastatic cases and correlated with an increase in *KMT2D* and *PIK3CA* alterations^16,28^. HPV-related PeCa were associated with a significantly lower TMB as compared to non-HPV-related PeCa, but presented with *FGF3* and *KMT2C* amplifications^13,24^. In non-HPV-related PeCa patients, *TERT* promoter mutations were more frequently observed as compared with HPV-related PeCa, which was further associated with a beneficial outcome^17^. On the contrary, HPV^-^ patients harboring *NOTCH1* mutations had a worse OS and PFS^20^.

Treatment-wise, PeCa are mostly managed using partial or total penectomy, Moh’s micrographic surgery, laser ablation, total glans resurfacing, radiation, or cisplatin-based chemotherapy. The latter regime, consisting of paclitaxel, ifosfamide, and cisplatin (TIP), can be used adjuvant, neoadjuvant or in palliative settings and presents with response-rates of about 65%^2,5,7,28^. However, only 8% have been reported in non-responders^2,5,7,28^, accordingly biomarkers identifying these non-responders are still urgently needed^29^. Hence, the evaluation of already established (targeted) therapeutic options as well as the identification of novel targets for the treatment of PeCa patients is still urgently required.

This study aimed to comprehensively characterize the (phospho-)proteome and secretome of patient- and xenograft-derived PeCa primary cultures in order to identify candidate biomarker as well as targetable signaling pathways. We subsequently analyzed the cytotoxicity of established chemotherapeutic drugs as well as (pre-clinical) pharmacological inhibitors in PeCa cells in vitro, which had been previously shown to be efficient in the treatment of germ cell tumors (GCT) and other urologic malignancies. By focusing on these preclinical models, the study provides a resource for putative future clinical efforts and strategies for the treatment of PeCa.

## Results

### Comprehensive profiling of the PeCa (phospho-)proteome and secretome reveals novel therapeutic targets

With the given rarity of PeCa and with the focus of literature on genomic and transcriptomic analyses, there is a lack of translational research and consequently alternative therapeutic options for PeCa. To overcome this, we chose a holistic proteomic approach by analyzing the proteome, secretome, and phosphokinome of PeCa cell lines (PeCa53, PeCa60, PeCa60Xen, PeCa60Xen^met^, PeCa65, PeCa70)^30^. By this, we deduced novel therapeutic targets and offer new potential treatment options for PeCa patients.

**Fig. 1:**
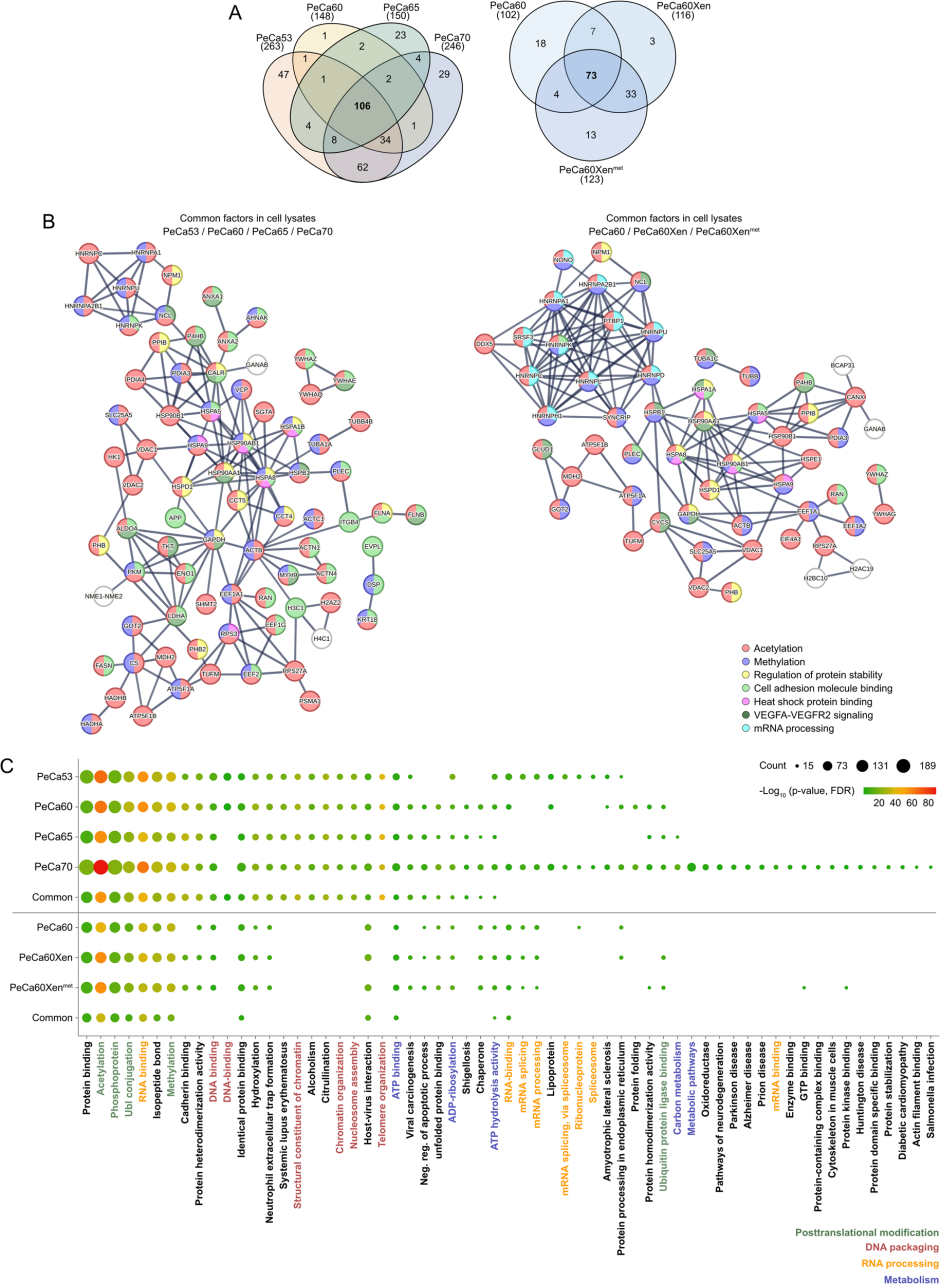
Identification of putative targets for the treatment of PeCa based on proteome analyses. **A** Venn diagrams showing commonly and exclusively translated proteins by PeCa53, PeCa60, PeCa65, and PeCa70 (left panel) and PeCa60, PeCa60Xen, PeCa60Xen^met^ (right panel; intensity >50,000,000). **B** Protein-protein interaction analyses (**B**) and **C** functional annotation analyses of the common proteins found in PeCa53, PeCa60, PeCa65, and PeCa70, as well as PeCa60, PeCa60Xen, and PeCa60Xen^met^. Data sets demarcated by a line, black dots indicating number of proteins, color indicating -log_10_(*p*-value), FDR-corrected using DAVID analyses.

Liquid chromatography coupled to mass-spectrometry (LC-MS) analyses revealed 106 proteins commonly shared in PeCa53, PeCa60, PeCa65, and PeCa70 cells (intensity > 50 × 10^6^) (Figs. 1A and S1A–C, and Data S1A). Additionally, 73 proteins were commonly measured in PeCa60 and related in vivo counterparts PeCa60Xen and PeCa60Xen^met^ (Dataset 2) (intensity > 50 × 10^6^) (Figs. 1A and S1A–C, and Data S1A). A STRING algorithm-based protein-interaction analysis of the most abundant proteins revealed that the proteins of both datasets were mainly involved in “acetylation”, “methylation”, “regulation of protein stability”, “cell adhesion molecule binding”, “heat shock protein binding” (e.g. HSP10, HSP27, HSP60, HSP70, HSP90), “VEGFA- / VEGFR2 signaling”, and “mRNA processing” (Fig. 1B). Functional annotation analysis using the DAVID algorithm supported this trend by showing the proteins’ involvement in posttranslational modifications (green) like “acetylation”, “methylation”, and ‘phosphoprotein’ (Fig. 1C). Additionally, detected proteins were linked to DNA packaging (red), but also RNA processing (yellow) and metabolic processes (blue) (Fig. 1C).

**Fig. 2:**
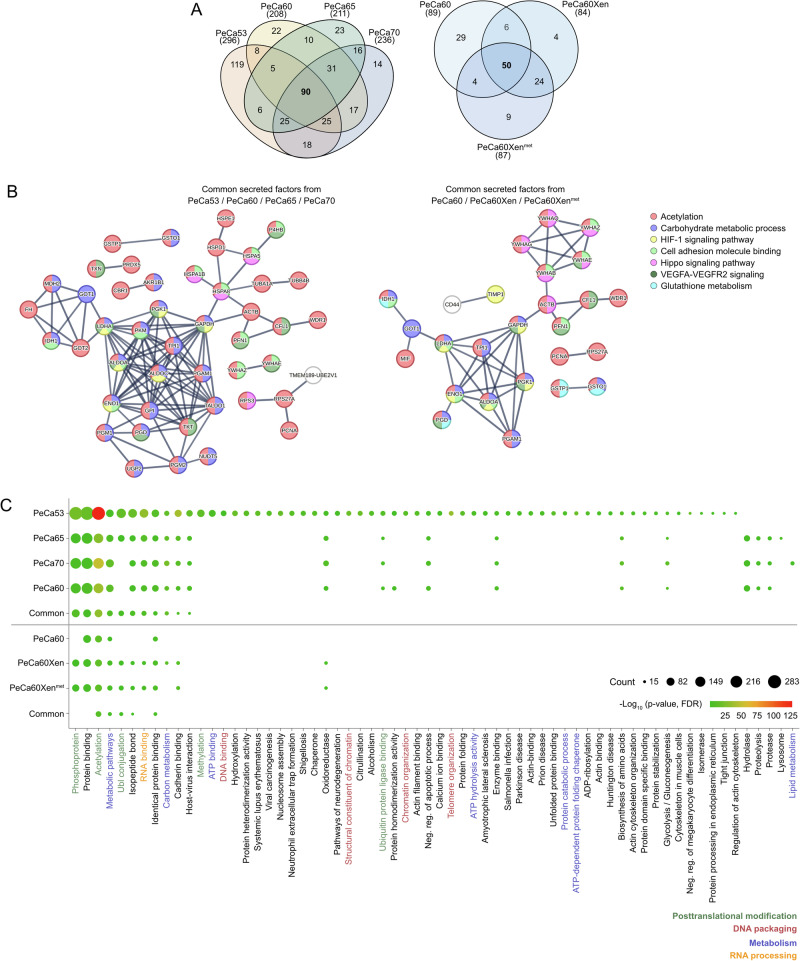
Identification of putative targets for the treatment of PeCa based on secretome analyses. **A** Venn diagrams illustrating commonly and exclusively secreted proteins by PeCa53, PeCa60, PeCa65, and PeCa70 (left panel) and PeCa60, PeCa60Xen, PeCa60Xen^met^ (right panel; intensity >50,000,000). **B** Protein-protein interaction analyses and **C** functional annotation analyses of the commonly secreted proteins found in PeCa53, PeCa60, PeCa65, and PeCa70, as well as PeCa60, PeCa60Xen, and PeCa60Xen^met^. Data sets demarcated by a line, black dots indicating number of proteins, color indicating -log_10_(*p*-value), FDR-corrected using DAVID analyses.

Next, secretome analyzes revealed 90 commonly secreted proteins in PeCa53, PeCa60, PeCa65, and PeCa70, whereas PeCa60 and its in vivo counterparts shared 50 commonly secreted factors (Figs. 2A and S1A–C, and Data S1B). As found prior in the cellular fraction of the proteome, a STRING-protein interaction analysis demonstrated that the secreted proteins play a role in “acetylation”, “cell adhesion molecule binding” and “VEGFA-VEGFR2 signaling”. Furthermore, secreted factors were involved in “carbohydrate metabolic and glutathione metabolism”, and the “HIF-1 and Hippo signaling pathway” (Fig. 2B). A DAVID annotation analysis supported these findings by showing involvement in “posttranslational modification” (green), “DNA packaging” (red), “metabolism” (blue) and “RNA processing” (yellow) (Fig. 2C). Prominently, heat shock proteins HSP10, HSP60, and HSP70 were also detected (Fig. 2C).

**Fig. 3:**
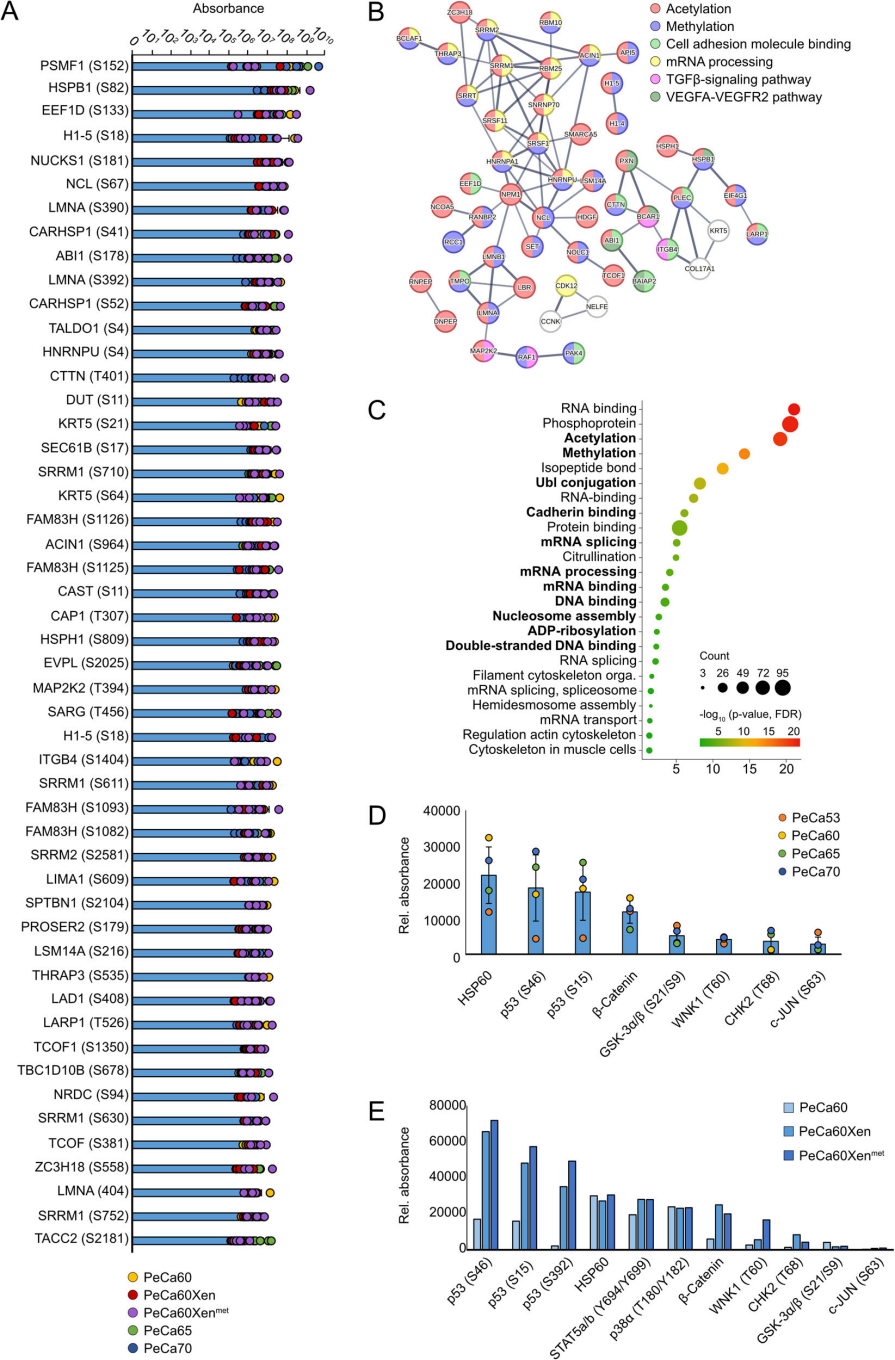
Identification of putative targets for the treatment of PeCa based on phospho-proteome analyses. **A** Top 50 of the most abundant phosphorylated proteins (dots indicating technical and biological replicates) as well as their **B** predicted protein-protein interaction and **C** functional annotation in PeCa60, PeCa60Xen, PeCa60Xen^met^, PeCa65, and PeCa70. Black dots indicating number of proteins, color indicating -log_10_(*p*-value), FDR-corrected using DAVID analyses. Phosphokinase array data of the most phosphorylated proteins observed in **D** PeCa53, PeCa60, PeCa65, and PeCa70 (dots indicating biological replicates) and **E** PeCa60, PeCa60Xen, PeCa60Xen^met^.

Finally, we analyzed the phospho-proteome of PeCa53, PeCa60, PeCa60Xen, PeCa60Xen^met^, PeCa65, and PeCa70 (Figs. 3A–E and S1C, and Data S1C). All PeCa cell lines showed high similarities based on the Top 50 most abundant phosphorylation sites (Fig. 3A). STRING and DAVID analyzes revealed that the most common phosphorylated factors were involved in epigenetic mechanisms (“acetylation”, “methylation”), cell adhesion (“cell adhesion molecule binding”, “cadherin binding”), “mRNA processing”, and “TGFβ- and VEGFA-VEGFR2 signaling pathways” (Fig. 3B, C). Again, heat shock proteins HSP27 and HSP105/110 (member of HSP70) were noted to be phosphorylated at S82 and S809, respectively (Data S1C). Performing a phospho-kinase array revealed that phosphorylation of HSP60 and P53 (S15, S46) were highly detectable in both datasets, with an even further increased phosphorylation of P53 (S15, S46, S392) in the in vivo counterparts of PeCa60 (Figs. 3D, E and S1D). Besides, high β-Catenin levels, as well as phosphorylation of GSK-3α/β (S21/S9), WNK1 (T60), CHK2 (T68), and c-JUN (S63) were noted in PeCa53, PeCa60, PeCa65, and PeCa70. Moreover, β-Catenin, p-CHK2 (T68), p-c-JUN (S63), p-STATa/b (Y694/Y699), p-p38α (T180/Y182), and p-WNK1 (T60), were enhanced in the xenograft and metastasis model (PeCa60Xen, PeCa60Xen^met^) (Figs. 3D, E and S1D). Of note, non-malignant fibroblasts previously indicated markedly lower phosphorylation levels at these sites as compared to GCT cells^31^. To further address the PeCa-specificity of the putative targets identified in the proteome and secretome, we incorporated data from non-malignant fibroblast cultures generated in our previous study for comparison (MPAF, LB-C18m, iLB-C1-30m, LB-C35m, and LB-C2-36m)^32^. As the fibroblast and PeCa samples were acquired in independent LC-MS experiments, direct quantitative comparisons of absolute protein abundances were not feasible. Therefore, we assessed the presence or absence of candidate proteins in the fibroblast datasets using an abundance threshold of >1,000,000 (Fig. S1E–H, and Data S1D). Within the proteome, 106 proteins were shared across all PeCa cultures (PeCa53, PeCa60, PeCa65, and PeCa70). Of these, 21 proteins were not detected in the fibroblast controls and can therefore be interpreted as PeCa-specific (Fig. S1E). As expected for a ubiquitously expressed housekeeping protein, GAPDH was detected in both fibroblasts and PeCa samples, whereas ACTB was detected exclusively in the PeCa proteome (Fig. S1E, G). A comparable pattern was observed in the secretome analysis, where ACTB was also among the 26 factors exclusively detected in PeCa-conditioned media (Fig. S1F, H). Notably, several proteins exclusively detected in the PeCa proteome were structural constituents of the chromatin, including H2AZ2, H2BC18, H3C1, and H4C1 (Fig. S1G, and Data S1D). The presence of these histone variants underscored the potential relevance of epigenetic dysregulation in PeCa and supports the rationale for evaluating HDACi as therapeutic strategies. Further comparison with fibroblasts revealed the involvement of Rho GTPase family members in PeCa cells (proteome: ACTB, ACTC1, DSP, H2AZ2, SFN, TUBA1A; secretome: ACTB, TUBA1A, TUBB4B), which are known regulators of cell-cycle progression^33^. Consequently, treatment with CDK4/6 inhibitors may represent a promising therapeutic strategy for PeCa. Across both proteome and secretome datasets, members of the HSP70 / HSP90 chaperone axis emerged as prominent candidates (Fig. S1G, H, and Data S1D). Of note, TUBA1A was identified exclusively in PeCa lysates and among the secreted factors, providing support for the current clinical use of taxanes (e.g. paclitaxel and docetaxel) in PeCa chemotherapy (Fig. S1G, H, and Data S1D).

As most clinical and translational studies investigating heat shock protein inhibition focus on HSP90, we further followed this established approach and selected pharmacological HSP90 inhibition as a representative strategy to target this pathway. In the second part of this study, we evaluated the cytotoxicity of various standard chemotherapeutic agents and pharmacological inhibitors. We included different alkylating agents, taxanes, topoisomerase inhibitors, and antimetabolites as standard chemotherapeutics in our cytotoxicity assays. Based on our previous work, additional potential targets and substance classes were investigated that had previously been identified as effective in GCTs and other urological entities.

### HDAC, CDK4/6, and HSP90 inhibition as therapeutic strategies for the treatment of PeCa

We included 15 different drugs, such as irinotecan (target: DNA packaging), JiB04 (target: methylation), romidepsin and quisinostat (target: acetylation), PRT4165 (target: ubiquitination), and 5-fluorouracil (5-FU) and chaetocin (target: metabolism), as well as anti-cancer agents routinely used in PeCa patients (cisplatin, paclitaxel, ifosfamide). We further included CDK4/6 inhibitors (palbociclib, ribociclib) due to promising results in GCT cell lines^34^, and HSP90 inhibitors (17-AAG, PU-H71) as not only protein-protein interaction analysis heavily revolved around HSP90-related proteins (Fig. S1E–H, and Data S1D), but also their efficacy for the treatment of GCT cells (Fig. S2). Briefly, HSP90α protein levels were markedly higher in GCT cell lines than in fibroblasts, while HSP70 expression was comparable (Fig. S2A). HSP90 inhibition with 17-AAG or PU-H71 reduced GCT cell viability (Fig. S2B), induced G2/M cell cycle arrest (Fig. S2C), and increased apoptosis induction (including PARP cleavage), with fibroblasts being less affected (Fig. S2D, E).

We evaluated the mRNA expression levels of potential drug target genes by qRT-PCR in PeCa and GCT cells as comparison (Fig. S3A). Similar or higher mRNA expression levels of *HDAC1/6*, *BRD4/7*, *RING1*, *SMARCA2/4*, *CDK4/6*, *HSP90AA1*, *HSPA1A/1B*, *CD24*, *CD274* / *PD-L1*, and *TACSTD2* / *TROP2* were noted in the PeCa cells as compared to the GCT cell lines TCam-2 (seminoma), 2102EP (embryonal carcinoma), GCT72 (yolk-sac tumor), and JAR (choriocarcinoma), as well as fibroblasts (MPAF, LB-C18m) (Fig. S3A). Expression of many of these targets was increased in the PeCa60Xen and PeCa60Xen^met^ cells as compared to their parental PeCa60 cells (Fig. S3B). These observations indicated that, based on the expression status, the modulation of the epigenetic landscape, as well as the inhibition of the cell cycle regulators CDK4/6 or the heat shock protein HSP90, represent promising therapeutic options for the treatment of PeCa.

**Fig. 4:**
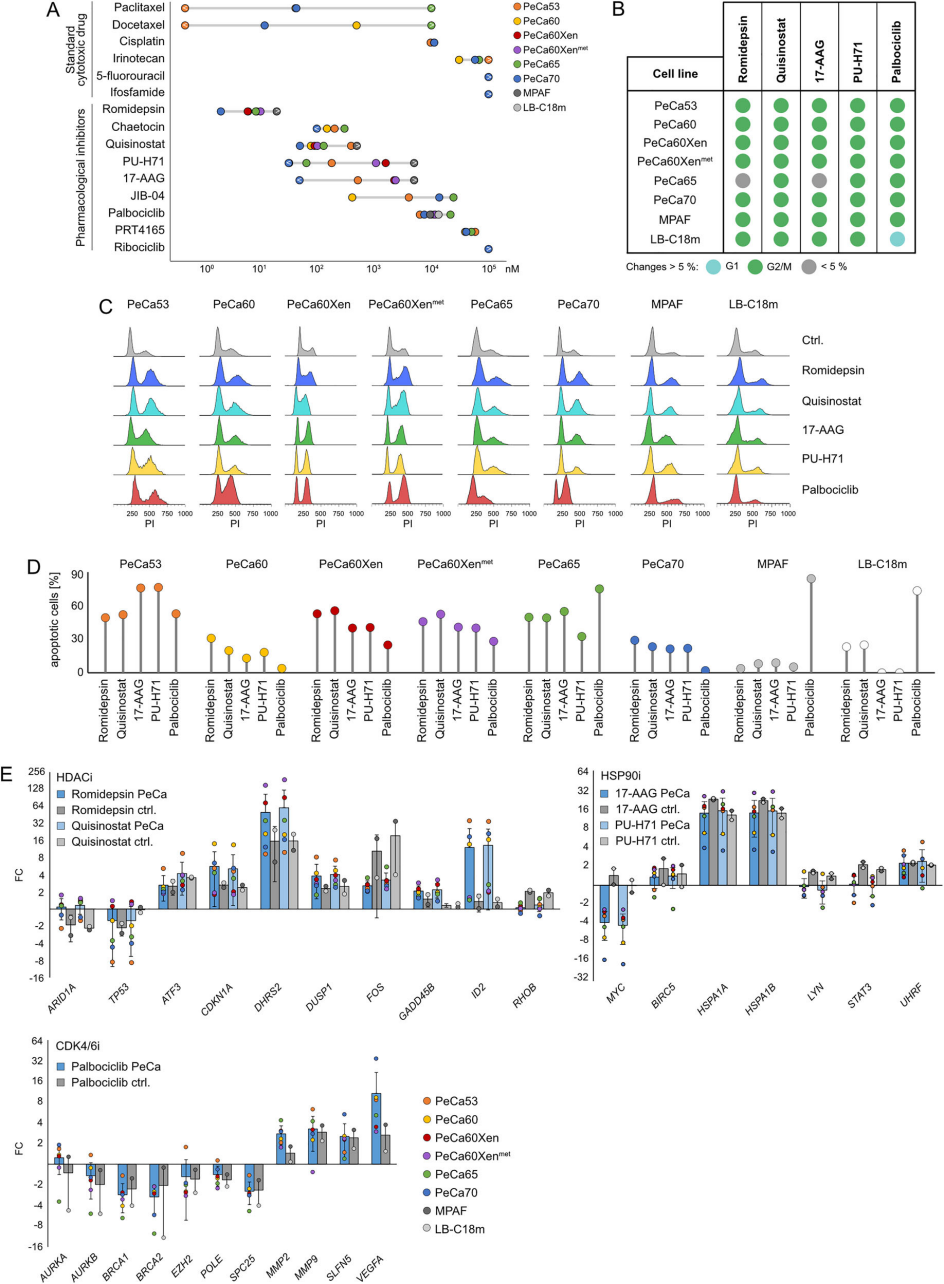
Screening therapeutic treatment options for PeCa. **A** Lollipop graph showing the LD_50_ values (72 h) of PeCa cells (PeCa53, PeCa60, PeCa60Xen, PeCa60Xen^met^, PeCa65, and PeCa70) and fibroblasts (MPAF and LB-C18m) treated with standard chemotherapeutic drugs (cisplatin, docetaxel, paclitaxel, 5-fluorouracil, ifosfamide, irinotecan), HDACi (romidepsin, quisinostat), HSP90i (17-AAG, PU-H71), HMTi (chaetocin), PRC1i (PRT4165), HDMi (JIB-04), or CDK4/6i (palbociclib, ribociclib). ‘>’ and ‘<’ denote that the estimated LD_50_ value exceeds or falls below the range of evaluated concentrations. **B** Changes in the cell cycle distribution and **C** raw cell cycle histograms of PeCa cells and fibroblasts treated with romidepsin, quisinostat, 17-AAG, PU-H71, or palbociclib for 16 h (LD_50 48 h_) as compared to the solvent control and as evaluated by propidium iodide staining with subsequent flow cytometry. **D** Apoptosis induction in PeCa cells and fibroblasts treated with romidepsin, quisinostat, 17-AAG, PU-H71, or palbociclib for 48 h (LD_50 48 h_) relative to the solvent control as evaluated by Annexin V-FITC / propidium iodide staining with subsequent flow cytometry. **E** mRNA expression levels of *ARID1A*, *ATF3*, *CDKN1A*, *DHRS2*, *DUSP1*, *FOS*, *GADD45B*, *ID2*, *RHOB* and *TP53* upon HDACi treatment (romidepsin, quisinostat), *BIRC5*, *HSPA1A*, *HSP1B1*, *LYN*, *MYC*, *STAT3*, and *UHRF* upon HSP90i treatment (17-AAG, PU-H71), and *AURKA/B*, *BRCA1/2*, *EZH2*, *MMP2/9*, *POLE*, *SLFN5*, *SPC25*, and *VEGFA* upon CDK4/6i treatment (palbociclib, ribociclib) in PeCa cells and fibroblasts (16 h, LD_50 48 h_) as compared to the solvent control. *ACTB* and *GAPDH* served as housekeeping genes.

Accordingly, cell viability assays were performed and revealed high sensitivities in low micromolar ranges towards HDAC, HSP90, and CDK4/6 inhibition in most PeCa cell lines (Figs. 4A and S4A–D). However, regarding the CDK4/6 inhibition, PeCa cells responded only towards the treatment with palbociclib (in a comparable concentration range as cisplatin), while ribociclib provoked only mild effects at tested concentrations (Figs. 4A and S3B, D).

Furthermore, docetaxel and paclitaxel showed a very heterogeneous and cell line dependent response among the PeCa cells, while 5-FU, ifosfamide and irinotecan were active only at high micromolar concentrations (Figs. 4A and S4A, C).

Subsequently, changes in the cell cycle distribution and apoptosis induction were evaluated upon treatment with the five most promising inhibitors (romidepsin, quisinostat, 17-AAG, PU-H71, palbociclib) in six PeCa cell lines (Fig. 4B–D). In comparison to the solvent control, treatment with most inhibitors resulted in accumulation of cells in the G2/M cell cycle phase in nearly all PeCa cells after 16 h (Fig. 4B, C). Also, an induction of apoptosis was observed under most treatment conditions compared to the solvent control after 48 h (Figs. 4D, S5A and S3B). Importantly, fibroblasts MPAF and LB-C18m indicated higher LD_50_ values upon treatment with romidepsin, quisinostat, 17-AAG, PU-H71, and palbociclib (Figs. 4A and S4B, D), thereby offering a therapeutic window. Even though changes in cell cycle distribution was also detected in fibroblasts upon treatment with these five most promising inhibitors (Fig. 4B, C), apoptosis was mostly induced to a lower extent as compared to the PeCa cells (Figs. 4D and S5A). Though, treatment with palbociclib for 48 h also resulted in fibroblasts in a high apoptosis induction (Figs. 4D and S5A). We further assessed the expression of apoptosis-related genes by qRT-PCR in PeCa60, PeCa60Xen, and PeCa60Xen^met^ cells following 24 h treatment with romidepsin, quisinostat, 17-AAG, PU-H71, or palbociclib (Fig. S5B). Pro-apoptotic genes associated with the intrinsic apoptosis pathway, including *APAF1*, *BAD*, *BAK1*, *BAX*, and *PMAIP1 / NOXA* were predominantly upregulated following treatment with either the HDACi or the HSP90i. *CASP7*, a central effector in both intrinsic and extrinsic apoptotic signaling, was downregulated upon HDACi treatment but upregulated in response to HSP90i (Fig. S5B). Components of the extrinsic apoptosis pathway (*CASP8*, *FADD*, and *FAS*) were generally reduced in PeCa60, PeCa60Xen, and PeCa60Xen^met^ cells following HDACi exposure, whereas only minor alterations were observed after HSP90i treatment (Fig. S5B). In contrast, treatment with the CDK4/6i palbociclib led to increased expression of *BAK1*, *BAX*, and *PMAIP1 / NOXA* (Fig. S5B). Anti-apoptotic genes, including *BCL2L1* and *BIRC5*, were downregulated following HDACi treatment, whereas *BCL2* and *BIRC5* were upregulated in HSP90i-treated cells (Fig. S5B). Summarizing up to this point, these findings indicated that treatment with HDACi, HSP90i, or CDK4/6i predominantly activated the intrinsic apoptotic signaling pathway in PeCa cells.

We tested for deregulations in expression of known effector molecules of each applied drug by qRT-PCR^34–39^. As shown before in GCT, urothelial, renal cell, and prostate carcinoma cells, increased expression of *ATF3*, *DHRS2*, *DUSP1*, *FOS*, and *ID2* was observed in PeCa cells upon treatment with the HDACi romidepsin and quisinostat (Fig. 4E)^35,36,38–40^. PeCa cells treated with the CDK4/6i palbociclib showed decreased levels of *AURKB*, *BRCA1/2*, *EZH2*, and *SPC25* expression, while expression of *MMP2/9*, *SLFN5*, and *VEGFA* was elevated, which is also comparable to previous observations in GCT, breast, and ovarian carcinoma cells (Fig. 4E)^34,41–43^. Treatment of PeCa cells with the HSP90i 17-AAG and PU-H71 increased the expression of *HSPA1A/1B* and *UHRF*, while diminishing the levels of *MYC*, which is in accordance with previously reported findings in cervix, lung, colon and prostate carcinoma or leukemia cells (Fig. 4E)^44,45^. Similar observations were also seen in fibroblasts treated with the HDACi, HSP90i, and CDK4/6i (Fig. 4E), an observation which we have described previously^34^.

Of note, as the HSP90i 17-AAG and PU-H71 are known to target the N-terminal domain of HSP90, eventually resulting in the activation of the tumor-promoting heat shock response, we further evaluated the cytotoxicity and molecular effects of a novel HSP90i (VWK147) targeting the C-terminal domain and, therefore, suspected not to endorse the heat shock response^45^.

Treatment of three PeCa cell lines (PeCa60, PeCa65, PeCa70) with VWK147 resulted in diminished cell viability and enhanced apoptosis induction (Fig. S6A, B). Similar to the previous observations of PeCa cells treated with 17-AAG or PU-H71, treatment with VWK147 resulted in enhanced expression levels of *HSPA1A/1B* (Fig. S4D). Besides, treatment with VWK147 also resulted in a diminished cell viability in (cisplatin-resistant) GCT cells (Fig. S2B). While cell cycle distribution was not affected in GCT cells (Fig. S2C), apoptosis induction was already detected after 24 h (Fig. S2D, E). These effects were only seen to a lesser extent in fibroblasts (Fig. S2B–E).

## Discussion

In this study, we addressed the current lack of translational research and targeted therapies for PeCa by employing a comprehensive proteomic strategy that encompassed analyzes of the cellular proteome, secretome, and the phospho-kinome across a panel of newly established PeCa cell lines (PeCa53, PeCa60, PeCa65, PeCa70), as well as in vivo-derived sublines (PeCa60Xen, PeCa60Xen^met^). Comparisons with non-malignant fibroblasts identified PeCa-specific chromatin-associated histone variants, factors from the Rho GTPase family known to influence progression through the cell cycle^33^, and heat shock proteins, thereby supporting the interreference with the epigenetic landscape, cell cycle progression, or heat shock response as putative therapeutic approaches for the treatment of PeCa by using HDAC, CDK4/6, and HSP90 inhibitors. While the detection of GAPDH in both PeCa cells and fibroblasts was expected for a ubiquitously expressed housekeeping protein, the exclusive detection of ACTB in the PeCa proteome and secretome suggested that cytoskeletal remodeling might be of relevance in PeCa cells, therefore validating the use of taxanes during the standard chemotherapeutic regime. Moreover, the presence of ACTB further supports the concept of (vesicle-mediated) unconventional protein secretion of intracellular proteins, likely driven by cellular stress^46^.

Although HDAC1/6 and CDK4/6 were not specifically detected in our LC-MS analyzes, several lines of evidence indicated that their functional relevance in PeCa was primarily driven by post-translational mechanisms rather than protein abundance. As such, the (phospho-)proteome analyzes revealed a strong enrichment of pathways linked to acetylation, cell cycle progression, and chromatin regulation, indicating a dependency on HDAC- and CDK-dependent pathways. Moreover, high *HDAC1/6* and *CDK4/6* mRNA expression levels were observed particularly in the xenograft-derived and metastatic PeCa sublines, thereby offering a therapeutic target for cisplatin-resistant PeCa patients. Another crucial cascade during cell cycle progression represents the Wnt/β-catenin signaling^47^. As such, in PeCa53, PeCa60, PeCa65 and PeCa70 we noted high β-catenin levels, which were even enhanced in the xenograft and metastasis model. Additionally, we observed GSK-3α/β inactivating phosphorylation at S21/S9, thereby preventing β-catenin phosphorylation and its subsequent ubiquitination and proteasomal degradation. Also Arya et al. noted significantly elevated protein levels of the Wnt ligand WNT4, and MMP7, CCND1, and c-MYC as targets of the Wnt-β-Catenin transcription activation in PeCa samples as compared to normal penile tissues^48^. While *MYC* copy number gains correlated with poor outcome in PeCa^49^, an inverse association was observed in a cohort of 41 PeCa patients regarding β-Catenin levels and tumor grading^50^. Additionally, *NUCKS1*, a factor controlling G1 to S cell cycle phase progression^51^, was detected by scRNA-seq within a PeCa basal stem cell subtype as well as a commonly phosphorylated protein found in our mass spectrometry data of PeCa60, PeCa60Xen, PeCa60Xen^met^, PeCa65, and PeCa70^52^.

Representing a major factor regulated by the ERK, RAF/MEK, Wnt/β-Catenin, JAK/STAT, PI3K/AKT/mTOR, and CDKN2A signaling cascades, CCND1 is a regulatory subunit of the CDK4/6 complex^53^. Hence, based on the findings observed in our study, we assume that the Wnt/ β-Catenin-mediated activation of CDK4/6 in PeCa could be implemented as a therapeutic approach. These findings were further supported by high levels of p38α (T180/Y182) and STAT5a/b (Y694/Y699)phosphorylation in PeCa60, PeCa60Xen, and PeCa60Xen^met^. Additionally, the scRNA-seq study by Song et al. further noted *RUNX1* to be highly elevated along the trajectory towards tumor cell differentiation, thereby representing factors relevant during PeCa progression^52^. RUNX1 was reported to regulate migration, invasion, and angiogenesis via the p38/MAPK pathway in human glioblastoma^54^. In line with our observations of p38α activation, RUNX1 phosphorylation was observed in PeCa60Xen cells.

Moreover, among the 8 most commonly phosphorylated factors, activation of WNK1 (T60), a positive regulator of the Wnt signaling pathway^55^, was observed in PeCa53, PeCa60, PeCa65, and PeCa70, as well as in the xenograft and metastasis model (PeCa60Xen, PeCa60Xen^met^). Also, Yang et al. observed *WNK1* missense mutations in 4% of the PeCa cases as compared to adjacent normal tissues^56^. Eventually, we assume that the targetability of CDK4/6 is of crucial interest for the treatment of PeCa cells.

Concerning the putative modification of the epigenetic machinery as a therapeutic strategy for the treatment of PeCa, we identified core components of the chromatin to be enriched in PeCa cells as compared to non-malignant fibroblasts. Also, Rogenhofer et al. noted that using a tissue microarray comprising 65 PeCa, six metastases, and 30 control tissues reduced levels of H3K4me1, H3K9me1/2, and H3K27me2/3, alongside increased H3K9me3 in PeCa, thereby supporting the evaluation of epigenetic therapies in (metastatic) PeCa patients^57^. Beyond that, limited data are available regarding the role of histone acetylation in PeCa. Moreover, DNA methylation profiling (Illumina Infinium Human Methylation 450K BeadChip) of PeCa indicated distinct clusters between PeCa and normal penile tissues, implying a hypermethylation phenotype associated with the malignancy^58,59^.

Prominently, our mass spectrometry-based studies of the (phospho-)proteome detected several heat shock proteins, such as HSP10, HSP27, HSP60, HSP70, or HSP90 in at least one of the evaluated datasets of the cellular fractions. One of the few mass spectrometry-based proteome analyses of PeCa revealed *inter alia,* that heat shock proteins were dominant in (HPV^+^) PeCa tissues as compared to (HPV^-^) healthy tissues^60^. Notably, our secretome profiling also revealed enhanced factors to be involved in metabolic and hypoxia-related pathways, including HIF-1 and Hippo signaling cascades. Again, HSP10, HSP60, and HSP70 were identified as secreted biomarkers. Regarding the relevance of heat shock proteins during PeCa progression, increased *HSP90B1* expression was found in a PeCa patient within the tumor differentiation branch as compared to the normal epithelial differentiation^52^, which also concurs with our findings of HSP90 as a putative therapeutic target.

So far, we identified heat shock proteins, posttranslational modifications, and cell cycle regulators to be of relevance in PeCa cells, thereby representing putative therapeutic targets. Subsequently, pharmacological inhibitors targeting HDAC (romidepsin, quisinostat), HSP90 (17-AAG, PU-H71), and CDK4/6 (palbociclib) were further included for functional cytotoxic assays. As such, diminished cell viability, induction of apoptosis, as well as an accumulation in the G2 / M cell cycle phase confirmed the cytotoxic efficacy. Regarding CDK4/6 inhibition, we observed treatment with palbociclib to be highly efficient for PeCa. However, in comparison to our previously reported observations in GCT cells^34^, treatment with ribociclib only resulted in a reduction of cell viability in high micromolar dosages in PeCa cells. Although both palbociclib and ribociclib target CDK4/6, this discrepancy is consistent with prior reports in other tumor entities and likely reflects the differences in intracellular drug accumulation (peak drug concentration, elimination half-life, volume of distribution, clearance), protein binding, or steady state rather than differences in *CDK4/6* expression^61^.

Also, treatment with the novel HSP90i C-terminal dimerization inhibitor VWK147 resulted in decreased cell viability, apoptosis induction, and disruption of the cell cycle distribution^45^. Treatment with this novel compound caused destabilization of typical HSP90 client proteins without inducing a heat shock response in urothelial carcinoma cells^62^. Furthermore, VWK147 was found to re-sensitize cisplatin-resistant urothelial carcinoma cells and, when combined with mTOR inhibition using Torin2, synergistically reduced cell viability in both cisplatin-sensitive and resistant cells; an effect not seen with the N-terminal HSP90i 17-AAG^62^. However, in contrast to the observations by David et al., *HSPA1B* and *HSPA1A* expression were upregulated in PeCa cells treated with VWK147 as compared to the solvent control^62^. Hence, a heat shock response still seemed to be provoked as it was seen with PeCa cells treated with the HSP90 N-terminal targeting inhibitors 17-AAG and PU-H71. Of note, in (cisplatin-resistant) urothelial carcinoma cells T24, treatment with 17-AAG as well as VWK147 resulted in a significantly decrease in *CDK4* expression^62^. In a phase I study, PU-H71 was administered *inter alia* in PeCa patients, eventually resulting in tumor regression of 20.8% (NCT01393509)^63^. Another clinical phase I/II trial is evaluating a miniature drug conjugate linking a HSP90 small molecule to a SN-38 cytotoxic payload (PEN-866) also in PeCa patients among other tumor entities^64^. Even though PEN-866 was well tolerated and indicated antitumor activity, the current status of this trial is unknown (NCT03221400).

Regarding the modulation of the epigenetic landscape, strategies combining HDACi with putative immunotherapeutic approaches, such as immune checkpoint inhibitors, adoptive T cell therapies, or antibody-drug-conjugates (ADC), remain relatively under-investigated in PeCa, resulting in limited available data. While in the PECOsq phase II basket trial, the combination of pembrolizumab with the HDACi vorinostat showed encouraging antitumor activity in SCC of the cervix and anus with unselected PD1 / PD-L1 status; the overall response rate was only 18% in PeCa. Additionally, the vorinostat dosage had to be reduced due to toxicity (NCT04357873)^65^. In our study, treatment with the HDACi romidepsin and quisinostat led to increased apoptosis and accumulation in the G2/M cell cycle phase in most PeCa cells, thereby representing promising therapeutic options. A currently active, not recruiting phase I/II study is evaluating the safety of combining the HDACi entinostat with PDS01ADC (a therapeutic vaccine targeting HPV 16 E6 / E7) and the IL-12-targeting immunocytokine M9241 in HPV-associated PeCa (NCT04708470)^66^. Similarly, a clinical phase II study demonstrated that the combination of the HDACi valproic acid and avelumab was safe to use for patients with Epstein-Barr virus-positive solid malignancies, such as PeCa (*n* = 3)^67^.

This study further evaluated the expression levels of a putative cell surface marker, which could be targeted using immune-therapeutic strategies. Specifically, higher mRNA levels of *CD24*, *CD274* / *PD-L1*, and *TACSTD2* / *TROP2* were observed in the PeCa cells as compared to the GCT cells or fibroblasts. At least since the FDA approval of the NECTIN4-targeting ADC enfortumab vedotin in combination with pembrolizumab for the treatment of patients with locally advanced or metastatic urothelial carcinoma in 2023^68^, the search for potentially targetable, tumor-specific cell surface markers has intensified. As such, high NECTIN4 levels correlated with reduced mortality in high-risk HPV^+^ PeCa, but had no substantial effects on tumor characteristics^69^. This is in accordance with the findings by Mink et al., who noted neither membranous nor cytoplasmic NECTIN4 positivity to be associated with metastasis-free survival, CSS, or OS^70^. With the exception of PeCa65, most PeCa cells presented with low *NECTIN4* expression; however, *NECTIN4* expression was elevated in the xenograft (PeCa60Xen) and the metastasis model (PeCa60Xen^met^) as compared to the parental cell type (PeCa60).

TROP2 is another cell surface protein, which can be targeted by an ADC (e.g. sacituzumab govitecan) and which has already been approved by the FDA for the treatment of patients suffering from unresectable/metastatic, hormone receptor-positive, HER2/ERBB2-negative breast cancer following endocrine-based therapy and chemotherapy^71^. Hence, based on immunohistochemical evaluation, high TROP2-positivity in high-risk HPV^+^ PeCa correlated with disease progression as compared to patients with low or intermediate TROP2 levels^69^. PeCa cells generally presented with higher *TACSTD2* / *TROP2* expression levels as compared to GCT cells, which were further enhanced in the xenograft and metastasis models (PeCa60Xen, PeCa60Xen^met^). Though Mink et al. did not observe a significant association of membranous or cytoplasmic TROP2 levels with the metastasis-free survival, CSS, or OS^70^. Additionally, even though mRNA expression of the membrane protein *CD24* was lower in PeCa cells as compared to EC cells, it could still represent a target using, for example immunotherapeutic approaches^72^.

Summarizing, through in-depth molecular characterization of the (phospho-)proteome and secretome of newly established PeCa cells, this study identified HDAC1/6, CDK4/6, and HSP90 as putative targets for the treatment of PeCa. As such, the pharmacological inhibition of those targets using romidepsin, quisinostat, 17-AAG, PU-H71, or palbociclib resulted in enhanced cytotoxicity in PeCa cells, consistent with the strong activation of stress- and cell-cycle-associated pathways, as reflected by elevated p53 phosphorylation, highlighting a potential alternative therapeutic approach. Moreover, this study offers CD24, HSP27, HSP60, HSP70, and TROP2 as novel targetable factors for the treatment of PeCa, though further validation is still required. Finally, while this study focused on single-agent treatments, our data provide a rationale for future combination strategies.

## Methods

### Cell culture

In this study, PeCa (PeCa53, PeCa60, PeCa60Xen, PeCa60Xen^met^, PeCa65 and PeCa70)^23^, GCT cell lines (TCam-2, 2102EP, GCT72, and JAR), and fibroblasts (MPAF, LB-C18m) were used. PeCa53 and PeCa60 represent primary cultures from the inguinal lymph node metastases, PeCa 65 and PeCa70 primary cultures from the primary tumors of PeCa patients treated at the Department of Urology at Saarland University. All patient-derived specimens were obtained from treatment-naïve patients, and model establishment occurred directly following tissue dissociation. PeCa60Xen is a primary culture derived from a murine PeCa60 renal-subcapsular xenograft tumor, while PeCa60Xen^met^ is a primary culture from a liver metastasis, which developed spontaneously in a murine PeCa60 renal-subcapsular xenograft model. PeCa, GCT, and fibroblast cells were maintained as a 2D monolayer under the conditions described in Table S1 A. Cell lines were tested for *Mycoplasma* contamination regularly as described previously^35^. Authenticity of all cell lines was evaluated by short tandem repeats profiles (Dr. Petra Böhme, Institute of Forensic Medicine Düsseldorf, University Hospital Düsseldorf, Düsseldorf, Germany). The ethics committee of the Heinrich Heine University Düsseldorf raised no concerns on utilizing GCT cell lines for in vitro experiments (votes 2018-178 and 2019-783 to D. N.). Generation of in vitro cultures from PeCa patient material was approved by the Ethics Committee of the Medical Association of Saarland (approval no. 42/17).

### Evaluation of cell viability

The evaluation of cell viability upon treatment via 2,3-bis-(2-methoxy-4-nitro-5-sulfophenyl)-5-[(phenylamino)carbonyl]-2H-tetrazolium (XTT)-assay (neoLab Migge GmbH, Heidelberg, Germany) was performed as described previously^73^. Briefly, 3 × 10^3^ cells were seeded on 96-well plates in 50 µl cell culture medium. The next day, cells were treated with inhibitors or chemotherapeutic compounds in quadruplicates (Table S1B). After 24 hours (h), 48 h, 72 h, and 96 h, 1 mg/ml XTT and 1.25 mM N-methyl dibenzopyrazine methyl sulfate (PMS; Sigma-Aldrich, Taufkirchen, Germany) were added for 4 h before evaluation of absorbance using a UV / VIS spectrometer (450 nm vs. 650 nm, iMark Microplate Reader; BioRad, Feldkirchen, Germany). LD_50_ doses were calculated using GraphPad Prism 9 (GraphPad Software, Boston, MA, USA).

### Flow cytometry

Apoptosis and cell cycle assays were performed as described previously^35^. Apoptosis assay was performed by Annexin V / propidium iodide (PI) staining after 48 h treatment with the inhibitors (LD_50 48h_) (Table S1B). Briefly, 9 × 10^4^ cells were seeded into 6-well plates before being treated with the inhibitors 24 h later. Upon enzymatic dissociation, cells were washed once in Annexin V-binding buffer (Miltenyi Biotec, Bergisch Gladbach, Germany) and were subsequently stained with 70 µl Annexin V-binding buffer containing 2.5 µl Annexin V-FITC (Miltenyi Biotec) and 15 µg PI (Sigma-Aldrich). After incubating in the dark at room temperature (RT) for 15 min, 500 µl Annexin V-binding buffer was added. Cell cycle assay was performed by PI staining after 16 h of treatment with the inhibitors (LD_50 48h_). Briefly, dissociated cells were washed once with PBS (Sigma-Aldrich) and then fixed with 70% ethanol/PBS. Subsequently, cells were washed twice with PBS and stained with 2 µg/ml PI and 200 µg/ml RNase A (Qiagen, Hilden, Germany) in PBS in the dark at RT for 10 min. 5.5 × 10^4^ cells per sample were measured using the MACSQuant Analyzer 10 (Miltenyi Biotec) and the results were analyzed using the Flowlogic software (Mentone, Victoria, Australia).

### Extraction of nucleic acids

DNA isolation was performed by phenol/chloroform/isoamyl alcohol (PCI) precipitation as described previously^32^. For RNA isolation, the RNeasy Mini Kit (Qiagen) was used according to the manufacturer’s protocol. Concentrations and quality of isolated nucleic acids were evaluated by NanoDrop measurement (ratios 260/280 nm, 260/230 nm).

### cDNA synthesis and quantitative RT-PCR

cDNA synthesis and qRT-PCT were performed as described previously^35,74^. For cDNA synthesis, 1 µg RNA in 12.5 µl RNase free H_2_O was mixed with 1 µl dNTP-Mix (10 mM), 1 µl Oligo(dT)_18_ primer (0.5 µg/µl), 4 µl RT buffer (5×), 1 µl Maxima H Minus Reverse Transcriptase (200 U/µl) and 0.5 µl RiboLock RNase Inhibitor (40 U/µl) (all Thermo Fisher Scientific, Schwerte, Germany). The samples were heated at 50 °C for 30 min and at 85 °C for 5 min using the S1000 Thermal Cycler (BioRad). For qRT-PCR, 7.35 ng cDNA per replicate was added to Luna Universal qPCR Master Mix (New England Biolabs, Frankfurt am Main, Germany) as well as oligonucleotide primers (see Table S1C). Each measurement was performed in technical triplicates and amplified using the 384-well C1000 Touch Thermal Cycler (BioRad).

### Western blot analysis

Determination of protein concentration and subsequent SDS-PAGE utilizing 20 µg of protein were performed as described previously^74,75^. Utilized antibodies are listed in Table S1D (Table S1D). Uncropped raw western blot images are presented in raw data file 1 (Raw data file 1).

### Quantitative mass spectrometric analysis of cell lysate and secreted proteins

Cell lysates and conditioned medium samples from PeCa cells (PeCa53, PeCa60, PeCa65, PeCa70) were prepared for liquid chromatography coupled to mass spectrometric analysis essentially as described earlier^76^. Briefly, cell lysis was carried out with lysis buffer containing 30 mM Tris-HCl; 2 M thiourea; 7 M urea; 4% 3-[(3-cholamidopropyl)dimethylammonio]-1-propanesulfonate (w/v) at pH 8.5 and proteins from conditioned medium samples were precipitated with trichloroacetic acid, washed with acetone and resuspended in lysis buffer mentioned above. After protein determination (Pierce's 660 nm assay, Thermo Fisher Scientific), 5 µg of protein was prepared for mass spectrometric by in-gel digestion with trypsin as described earlier^76^. Finally, peptides from conditioned medium were purified by solid phase extraction (HLB material, µ-elution plate, Waters) and 500 ng peptides from conditioned medium and lysate samples were resuspended in 0.1% (v / v) trifluoroacetic acid (TFA) and subjected to liquid-chromatography coupled mass spectrometric analysis. An Ultimate 3000 rapid separation liquid chromatography system (Thermo Fisher Scientific) was used to first trap the peptides on an Acclaim PepMap100 C18 column (2 cm length, 3 μm particle size, 100 Å pore size, 75 μm inner diameter; Thermo Fisher Scientific) and then to separate them by a 2 h gradient on a C18 PepMapRSLC column (Acclaim 25 cm length, 2 μm particle size, 100 Å pore size, 75 μm inner diameter, Thermo Fisher Scientific) as described previously^77^. Separated peptides were then injected via a nano-source interface into a Fusion Lumos Tribrid mass spectrometer (Thermo Fisher Scientific) operated in positive mode. The mass spectrometer was equipped with a field asymmetric ion mobility spectrometer device. PeCa samples were analyzed in a data-dependent mode: first precursor spectra were recorded in the orbitrap (resolution 120,000, scan range 375–1500 *m*/*z*, maximum injection time 60 ms, AGC target 400000, charge states 2–7), then, precursors were isolated (quadrupole isolation window 1.6) fragmented by higher energy collisional dissociation (30% collision energy, 5% stepped collision energy) and fragment spectra recoded in the ion trap (scan rate rapid, maximum injection time 35 ms, AGC target 10000). Cycle time was 1 s for alternating compensation voltages of −40 V, −55 V and −70 V. Dynamic exclusion was set to 1 min. Protein identification and precursor ion-based quantification were carried out with Proteome Discoverer 2.4.1.15 with the Sequest HT search engine. Homo sapiens sequences from the UniProt KB proteome section (UP000005640, downloaded on 12th January 2023, 81837 entries) and additional potential contaminant sequences from MaxQuant 1.6.17.0 were used for the search. Mass deviation was set to 20 ppm (precursor) and 0.5 Da (fragment). Carbamidomethyl was set as fixed and protein N-terminal acetylation, protein N-terminal methionine loss and methionine oxidation as variable modification. Precursor-based label-free quantification was enabled and normalization based on total peptide amount. False discovery rate (FDR) was set to 1% on peptide and protein level (Percolator).

PeCa60, PeCa60Xen, and PeCa60Xen^met^ were analyzed in a data-independent mode at a compensation voltage of −50 V: first precursor spectra were recorded in the orbitrap (resolution 60,000, scan range 380–985 *m*/*z*, maximum injection time 100 ms, AGC target 400000, charge states 2–7), then, precursors were selected within 10 *m*/*z* isolation windows by the quadrupole in the mass range of 380–980 *m*/*z*. Precursors were fragmented by higher energy collisional dissociation (30% collision energy, 5% stepped collision energy) and fragment spectra recorded in the orbitrap (scan range of 145–1450 *m*/*z*, resolution of 15,000, automatic gain control target 100000, maximum injection time 40 ms, centroid mode). The used cycle time was 3 s. Raw data were further processed with Dia-NN 1.8.2 for peptide and protein identification and quantification using standard parameters if not stated otherwise. Oxidation was considered as additional variable modification, and a spectral library generated by Dia-NN based on 81837 homo sapiens sequences (UniProt KB proteome: UP000005640) and additional potential contaminant sequences from MaxQuant 2.1.0.0. Proteins with Q.Value and Global.PG.Q.Value < 0.01 as well as PG.Q.Value < 0.05 were removed. For all experiments potential contaminants were removed, and only proteins considered which were identified with at least two different peptides.

### Quantitative mass spectrometric analysis of the phospho-proteome

Cells were trypsinized, washed with PBS, and lysed in RIPA buffer (Thermo Fisher Scientific) supplemented with protease inhibitors (cOmplete Mini, EDTA-free; Roche, Mannheim, Germany) and phosphatase inhibitors (PhosSTOP, Roche). Lysates were incubated on ice for at least 30 min and subsequently centrifuged at high speed for 20–30 min at 4 °C. The supernatant was collected and used for further processing. Protein concentrations were determined using the DC Protein Assay (Bio-Rad) in a 96-well microplate format and measured with a Tecan plate reader. A total of 400 µg protein was adjusted to a final volume of 112.5 µl using lysis buffer. Proteins were reduced by addition of DTT (final concentration 5 mM) and incubated for 20 min at 56 °C, followed by alkylation with CAA (final concentration 15 mM) for 20 min at 37 °C. Protein digestion was performed using the Protein Aggregation Capture (PAC) method with magnetic amine beads (MagReSyn), using a 5:1 bead-to-protein ratio. Samples were incubated with sequencing-grade trypsin (Promega, Walldorf, Germany) at a 1:50 enzyme-to-substrate ratio in 25 mM ammonium bicarbonate buffer and processed on a KingFisher system using a standard 4.5-h protocol. After digestion, peptides were acidified with TFA to a final concentration of 0.1% and desalted using Pierce Peptide Desalting Spin Columns (Thermo Fisher Scientific) according to the manufacturer’s protocol. Phosphopeptides were enriched using a combination of Zr-IMAC HP and Ti-IMAC HP magnetic beads (MagReSyn) in loading buffer containing 80% acetonitrile (ACN), 5% TFA, and 1 M glycolic acid. Enrichment was carried out on the KingFisher system using a dedicated phosphopeptide protocol. Elution was performed with 1% ammonium hydroxide, and eluates were vacuum-dried at 45 °C. Prior to mass spectrometry, samples were reconstituted in 0.1% TFA with 1% ACN, spiked with indexed retention time (iRT; Biognosys, Schlieren, Switzerland) peptides, centrifuged, and cleared of residual beads using a magnetic rack.

Peptides were analyzed using data-independent acquisition (DIA) on a Thermo Scientific Exploris 480 mass spectrometer coupled to a high-performance liquid chromatography system equipped with 110 cm µPac Neo analytical column (Thermo Fisher Scientifc). Chromatographic separation was achieved using a linear gradient over 120 min (this includes equilibration and washing steps), ramping from 5% to 40% acetonitrile in 0.1% formic acid at a flow rate of 200 nl/min. The DIA method included 40 variable windows covering a mass range of 400–1400 *m*/*z*, with narrower isolation widths in regions of higher precursor density. Full MS1 scans were acquired at a resolution of 120,000 with the maximum injection time Mode Set to Auto and AGC Target set to Standard, followed by MS2 scans at a resolution of 30,000. The AGC target was set to 1e6 both for MS1 and MS2. Normalized collision energy (NCE) was set to 30 for HCD fragmentation.

Data were processed with Spectronaut (version 19.7; Biognosys) using the directDIA+ (Deep) workflow. Carbamidomethylation of cysteine was set as a fixed modification, and phosphorylation (STY), methionine oxidation, and N-terminal acetylation were considered as variable modifications. Peptide identification and quantification were performed at 1% FDR on precursor, peptide, and protein levels. For phosphosite localization, a probability cutoff of 0.7 was applied. Quantification was based on MS2 area under the curve (AUC), with interference correction enabled and top 3 peptide precursors per protein group used for label-free quantification.

### Phospho-kinase array

Protein concentration of each sample was measured by using the Pierce BCA protein assay kit according to the manufacturer’s protocol (Thermo Fisher Scientific). The Proteome Profiler Human Phospho-Kinase Array Kit (R&D Systems via Bio-Techne, Wiesbaden, Germany) was performed using 350 µg protein lysate for each sample according to the manufacturer’s manual. Densitometric evaluation was analyzed by means of Image J with the Protein Array Analyzer plugin^78,79^.

### Online analysis tools and statistical analyses

LC-MS data were visualized by principal component analyzes (PCA) using “PCAGO” (https://pcago.bioinf.uni-jena.de/)^80^ and “Venny” for Venn diagrams (https://bioinfogp.cnb.csic.es/tools/venny)^81^. Molecular functions were predicted by “DAVID Functional Annotation Tool” (https://david.ncifcrf.gov) using “UP_KW_PTM”, “GOTERM_BP_DIRECT”, “GOTERM_MF_DIRECT” and “KEGG_PATHWAY”^82^ and visualized in enrichment plots using “ImageGP” (https://www.bic.ac.cn/ImageGP)^83^, while protein interactions were analyzed using ‘STRING’ (network edges sorted for confidence, highest confidence 0.9, hide disconnected nodes in the network) (https://string-db.org/)^84^.

Differences between groups were analyzed using a two-tailed Student’s *t*-test and highlighted by asterisks (^*^p < 0.05).

## Supplementary information

Supplementary information

Supplementary data

## Data Availability

All data generated or analyzed during this study are included in the published article and its supplementary information files. Additionally, proteome datasets produced in this study are available via ProteomeXchange (PXD071333).
