# Supplementary material for: Targeting histone deacetylation, cell cycle regulators and heat shock proteins as novel therapeutic strategies for penile cancers
Source: NPJ Precis Oncol. 2026 Mar 31;10:140. doi: 10.1038/s41698-026-01391-4 (PMC13043893; doi:10.1038/s41698-026-01391-4)

Targeting histone deacetylation, cell cycle regulators and heat shock proteins as novel therapeutic strategies for penile cancers

Lara Marson[1*], Margaretha A. Skowron[1*], Pailin Pongratanakul[1], Mara Kotthoff[1], Gereon Poschmann[2], Hanibal Bohnenberger[3], Alexa Stephan[1], Meike M. Watolla[1], Elvira Mukinovic[1], Kai Stühler[2], Thomas Kurz[4], Hiresh Ayoubian[5], Johannes Linxweiler[5], Kerstin Junker[5], Daniel Nettersheim[1,6+]

[1] Department of Urology, Urological Research Laboratory, Translational UroOncology, Medical Faculty and University Hospital Düsseldorf, Heinrich Heine University Düsseldorf, Germany
[2] Molecular Proteomics Laboratory (MPL), Biological and Medical Research Center (BMFZ), Medical Faculty and University Hospital Düsseldorf, Heinrich Heine University, Germany
[3] Institute of Pathology, University Medical Center Göttingen, Germany.
[4] Department of Pharmaceutical and Medical Chemistry, Heinrich Heine University Düsseldorf, Düsseldorf, Germany
[5] Department of Urology and Pediatric Urology, Saarland University, Homburg, Germany
[6] Center for Integrated Oncology Aachen Bonn Cologne Düsseldorf (CIO ABCD), Düsseldorf, Germany

* contributed equally

Supplementary material

[+] corresponding author:

Prof. Dr. Daniel Nettersheim
Medical Research Center I
Urological Research Laboratory
Translational UroOncology
Medical Faculty and University Hospital Düsseldorf
Heinrich Heine University
Moorenstraße 5
40225 Düsseldorf
Germany
E-Mail: Daniel.Nettersheim@med.uni-duesseldorf.de
Phone: +49 211 81 06731

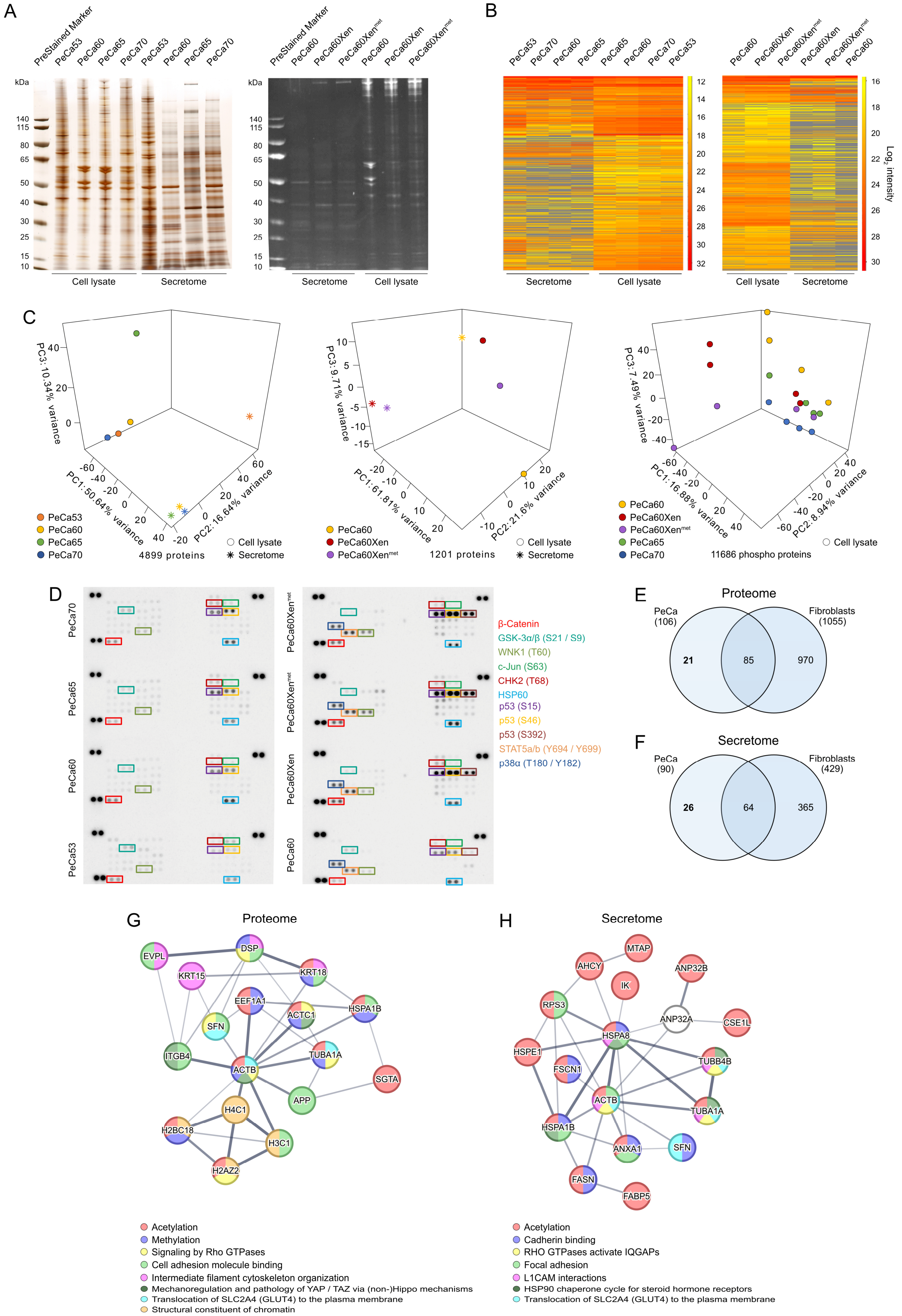
A
PreStained Marker
PeCa53
PeCa60
PeCa65
PeCa70
PeCa53
PeCa60
PeCa65
PeCa70
kDa
140
115
80
65
50
40
30
25
15
10
Cell lysate
Secretome
PreStained Marker
PeCa60
PeCa60Xen
PeCa60Xen^met
PeCa60
PeCa60Xen
PeCa60Xen^met
Secretome
Cell lysate
B
Secretome
Cell lysate
Cell lysate
Secretome
Log2 intensity
C
PC1: 50.64% variance
PC2: 16.64% variance
PC3: 10.34% variance
PeCa53
PeCa60
PeCa65
PeCa70
4899 proteins
Cell lysate
Secretome
PC1: 61.81% variance
PC2: 21.6% variance
PC3: 9.71% variance
PeCa60
PeCa60Xen
PeCa60Xen^met
1201 proteins
Cell lysate
Secretome
PC1: 16.88% variance
PC2: 8.94% variance
PC3: 7.49% variance
PeCa60
PeCa60Xen
PeCa60Xen^met
PeCa65
PeCa70
11686 phospho proteins
Cell lysate
D
PeCa70
PeCa65
PeCa60
PeCa53
PeCa60Xen^met
PeCa60Xen^met
PeCa60Xen
PeCa60
β-Catenin
GSK-3α/β (S21 / S9)
WNK1 (T60)
c-Jun (S63)
CHK2 (T68)
HSP60
p53 (S15)
p53 (S46)
p53 (S392)
STAT5a/b (Y694 / Y699)
p38α (T180 / Y182)
E
Proteome
PeCa (106)
Fibroblasts (1055)
21
85
970
F
Secretome
PeCa (90)
Fibroblasts (429)
26
64
365
G
Proteome
EVPL
DSP
KRT15
KRT18
EEF1A1
ACTC1
HSPA1B
SFN
ITGB4
TUBA1A
ACTB
SGTA
APP
H4C1
H2BC18
H3C1
H2AZ2
Acetylation
Methylation
Signaling by Rho GTPases
Cell adhesion molecule binding
Intermediate filament cytoskeleton organization
Mechanoregulation and pathology of YAP / TAZ via (non-)Hippo mechanisms
Translocation of SLC2A4 (GLUT4) to the plasma membrane
Structural constituent of chromatin
H
Secretome
MTAP
AHCY
ANP32B
IK
RPS3
ANP32A
CSE1L
HSPA8
HSPE1
FSCN1
TUBB4B
ACTB
TUBA1A
HSPA1B
ANXA1
SFN
FASN
FABP5
Acetylation
Cadherin binding
RHO GTPases activate IQGAPs
Focal adhesion
L1CAM interactions
HSP90 chaperone cycle for steroid hormone receptors
Translocation of SLC2A4 (GLUT4) to the plasma membrane

Figure S1:
A) Protein extraction quality control for liquid chromatography-mass spectrometry (LC-MS / MS) as indicated by silver staining of cell lysates and supernatants. B) Hierarchical cluster analysis of proteins identified in the cell lysates or secretome of PeCa53, PeCa60, PeCa60Xen, PeCa60Xen^{met}, PeCa65, and PeCa70 cells as measured by LC-MS / MS. C) Principal component analyses of the proteome and secretome data (left and middle), as well as phospho-proteome data (right). D) Raw human phospho-kinase arrays of various cell lysates (PeCa53, PeCa60, PeCa60Xen, PeCa60Xen^{met}, PeCa65, and PeCa70 cells). Corresponding membrane layout with the most prominent dots (marked in color) was used for quantification using ImageJ as shown in Fig. 3 D, E. Venn diagrams illustrating factors exclusively found in the E) proteome and F) secretome of PeCa cells after deduction of factors detected in fibroblasts (MPAF, LB-C18m, iLB-C1-30m, LB-C35m, and LB-C2-36m). Predicted protein-protein interactions of factors found exclusively in the G) proteome and in the H) secretome of PeCa cells.

A

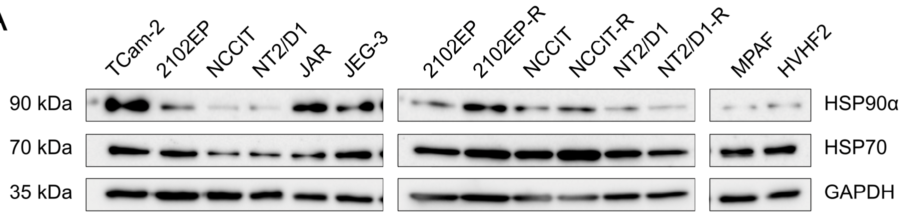

B

Cell line	17-AAG	PU-H71	VWK-147
TCam-2	> 10	0.07	1.32
2102EP	0.93	0.11	1.22
2102EP-R	4.28	0.23	2.32
NCCIT	4.99	0.33	3.04
NCCIT-R	2.38	0.46	2.12
NT2/D1	> 10	0.32	2.15
NT2/D1-R	1.43	0.16	0.99
JAR	> 10	> 0.5	7.34
JEG-3	1.53	> 0.5	2.64
MPAF	> 10	> 0.5	6.08
HVHF2	> 10	> 0.5	6.37

$LD_{50\ 48\ h}$
< 0.5 µM
0.5 - 10 µM
> 10 µM

C

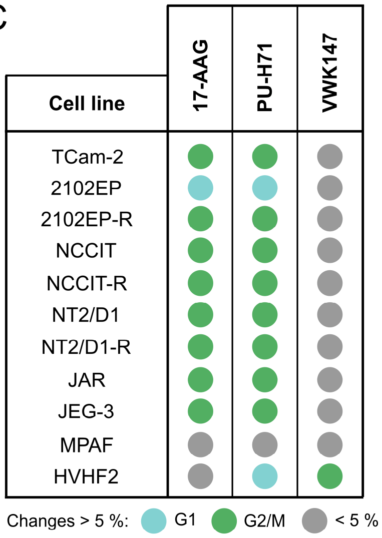

D

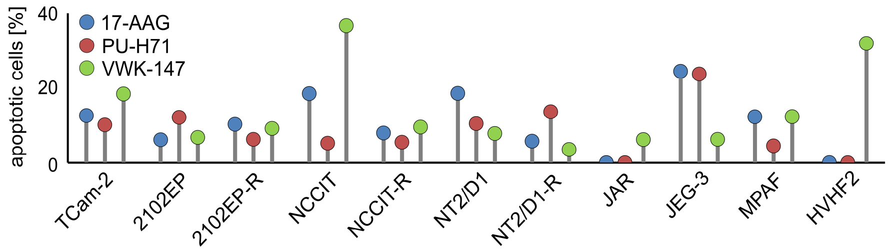

E

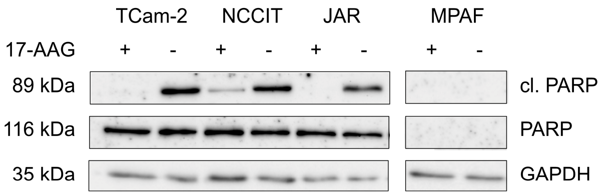

Figure S2:
A) Western blot analyses showing HSP90α and HSP70 protein levels of (cisplatin-resistant) GCT cell lines (TCam-2, 2102EP, NCCIT NT2/D1, JAR, JEG-3) and fibroblasts (MPAF, HVHF2). GAPDH served as a loading control. B) Calculated LD_{50} values in (cisplatin-resistant) GCT cells (TCam-2, 2102EP, NCCIT NT2/D1, JAR, JEG-3) and fibroblasts (MPAF, HVHF2) after treatment with 17-AAG, PU-H71, and VWK-147 for 48 h. C) Cell cycle distribution and D) apoptosis induction in (cisplatin-resistant) GCT cells (TCam-2, 2102EP, NCCIT NT2/D1, JAR, JEG-3) and fibroblasts (MPAF, HVHF2) after treatment with 17-AAG (5 µM), PU-H71 (5 µM), and VWK147 (2.5 µM) for 24 h. E) Western blot analyses indicating PARP cleavage and total PARP protein levels in TCam-2, NCCIT, JAR, and MPAF cells upon treatment with 5 µM 17-AAG for 20 h.

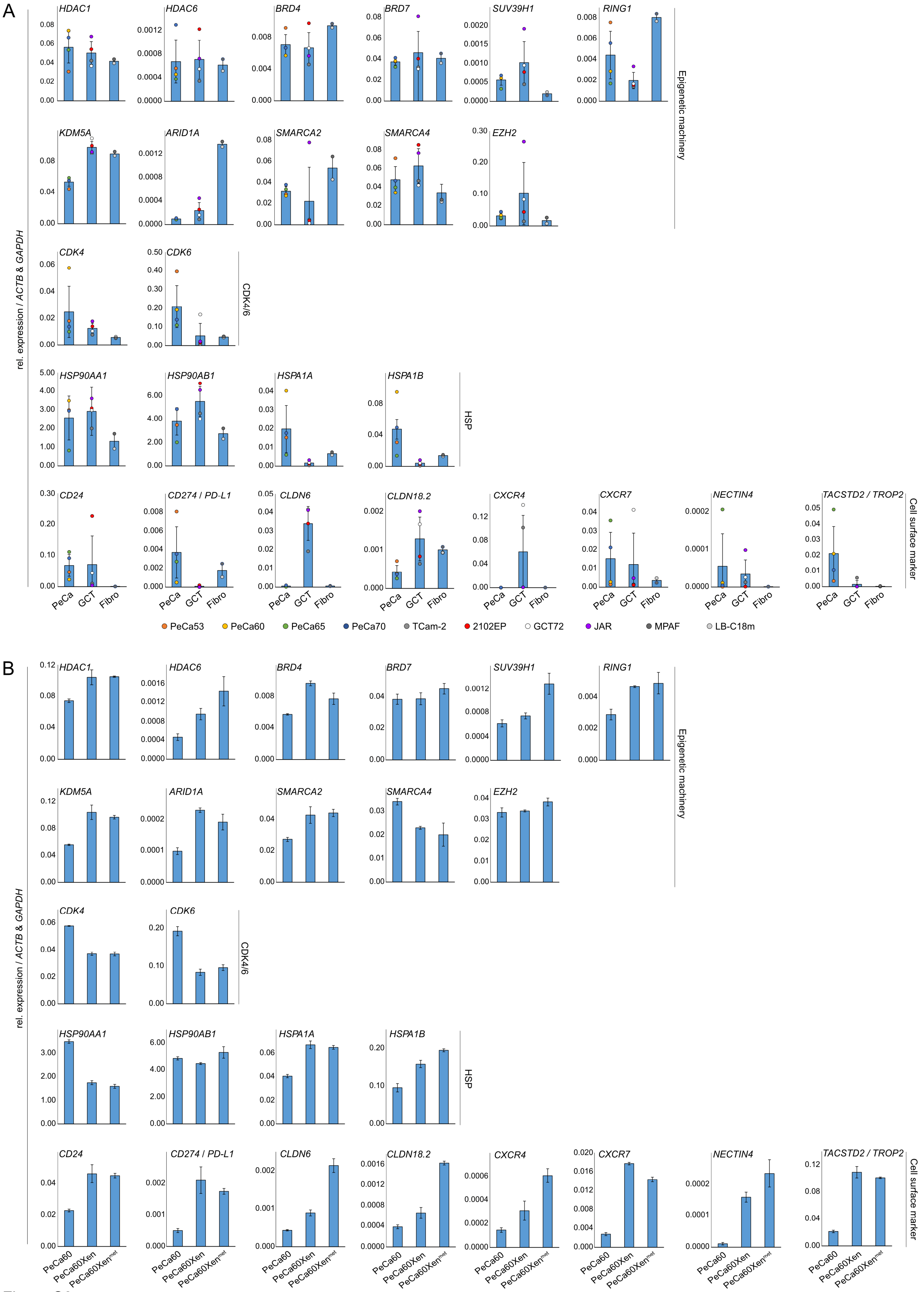

Figure S3:
Basal mRNA expression levels of putative therapeutic targets (*HDAC1 / 6*, *BRD4 / 7*, *SUV39H1*, *RING1*, *KDM5A*, *ARID1A*, *SMARCA2 / 4*, *EZH2*, *CDK4 / 6*, *HSP90AA1 / AB1*, *HSPA1A / 1B*, *CD24*, *CD274 / PD-L1*, *CLDN6*, *CLDN18.2*, *CXCR4 / 7*, *NECTIN4*, and *TACSTD2 / TROP2*) in A) PeCa cells (PeCa53, PeCa60, PeCa65, PeCa70), GCT cells (TCam-2, 2102EP, JAR, GCT72), and fibroblasts (MPAF, LB-C18m), as well as in B) PeCa60, PeCa60Xen, and PeCa60Xen^{met} cells. *ACTB* and *GAPDH* served as housekeeping genes.

A

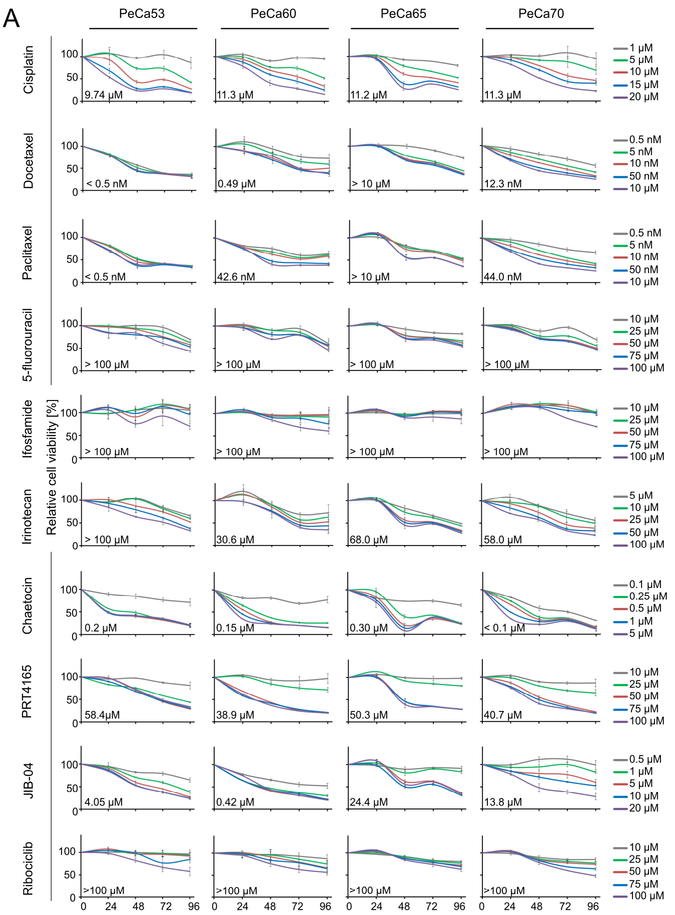

B

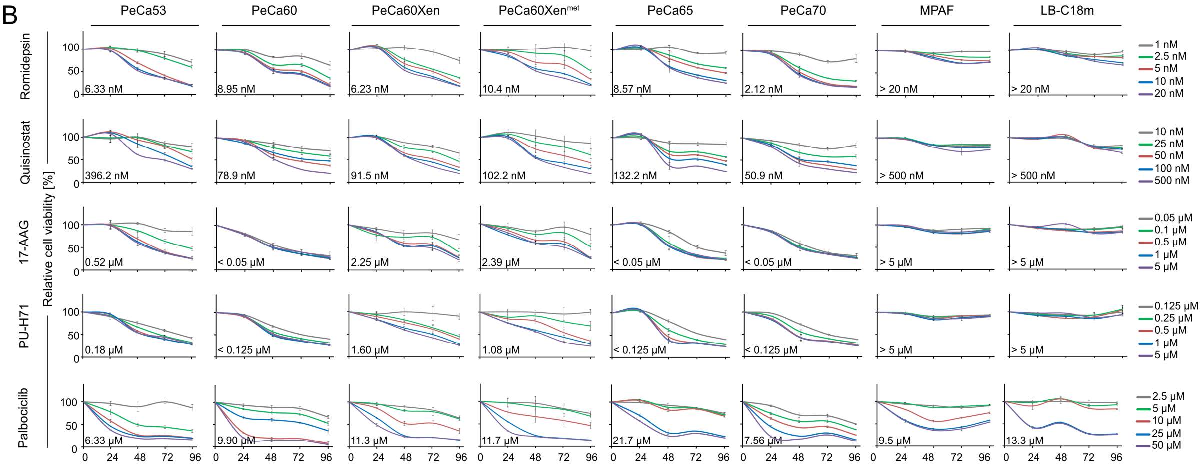

C

Cell line	Cisplatin	Docetaxel	Paclitaxel	5-FU	Ifosfamide	Irinotecan	Ribociclib	Chaetocin	PRT4165	JIB-04
PeCa53	8.86	0.01	< 0.01	> 100	> 100	> 100	> 100	0.59	> 100	18.6
PeCa60	18.1	> 10	0.38	> 100	> 100	> 100	> 100	0.24	57.4	2.34
PeCa65	11.7	> 10	> 10	> 100	> 100	53.5	> 100	0.20	64.4	18.0
PeCa70	16.9	0.45	0.84	> 100	> 100	> 100	> 100	0.12	68.9	19.1

$LD_{50\ 48\ h}$ < 1 µM 1 - 50 µM > 50 µM

D

Cell line	Romidepsin	Quisinostat	17-AAG	PU-H71	Palbociclib
PeCa53	0.02	0.78	6.33	5.01	6.52
PeCa60	0.02	0.51	2.11	2.56	11.5
PeCa60Xen	0.02	0.81	4.54	7.62	12.6
PeCa60Xen^{met}	0.02	0.38	7.27	5.50	15.3
PeCa65	0.02	0.14	2.68	0.75	19.5
PeCa70	0.01	0.14	0.45	0.65	6.66
MPAF	> 0.02	> 0.5	> 5	> 5	14.1
LB-C18m	> 0.02	> 0.5	> 5	> 5	16.0

$LD_{50\ 48\ h}$ < 1 µM 1 - 50 µM > 50 µM

Figure S4:
A) XTT cell viability assays of PeCa53, PeCa60, PeCa65, and PeCa70 cells treated with indicated chemotherapeutic compounds and inhibitors for 24 - 96 h (n = 4). LD_{50} values after 72 h are included in the graphs. B) XTT cell viability assays of PeCa53, PeCa60, PeCa60Xen, PeCa60Xen^{met}, PeCa65, PeCa70, MPAF, and LB-C18m cells with indicated chemotherapeutic compounds and inhibitors for 24 - 96 h (n = 4). C) and D) Calculated LD_{50} values after 48 h for the corresponding graphs shown in (A) and (B), respectively. Corresponding to the data shown in Fig. 4 A.

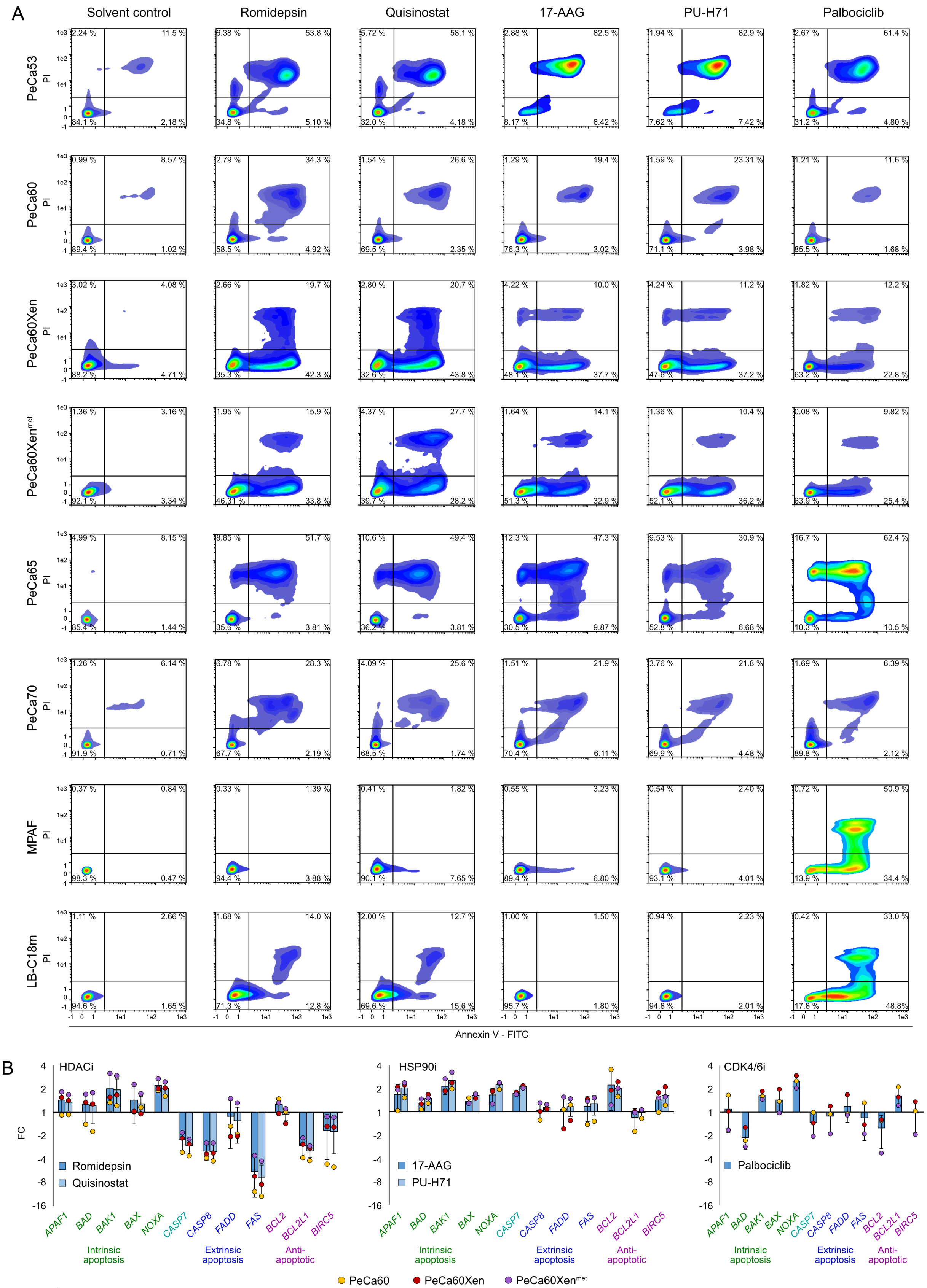

Figure S5:
A) Scatter plots showing Annexin V-FITC and propidium iodide staining with subsequent flow cytometry in PeCa cells (PeCa53, PeCa60, PeCa60Xen, PeCa60Xen^{met}, PeCa65, PeCa70) and fibroblasts treated with the corresponding $LD_{50\ 48\ h}$ concentrations of romidepsin, quisinostat, 17-AAG, PU-H71, or palbociclib for 48 h. Corresponding to the data shown in Fig. 4 D. B) mRNA expression levels of factors related to the intrinsic (*APAF1*, *BAD*, *BAK1*, *BBC3 / PUMA*, *CASP7*, *NOXA*) and extrinsic (*CASP7*, *CASP8*, *FADD*, *FAS*) apoptosis cascade or serving as inhibitors of apoptosis (*BCL2*, *BCL2L1*, *BIRC5*) in PeCa60, PeCa60Xen and PeCa60Xen^{met} cells treated with the corresponding $LD_{50\ 48\ h}$ concentrations of romidepsin, quisinostat, 17-AAG, PU-H71, or palbociclib for 24 h. *ACTB* and *GAPDH* served as housekeeping genes.

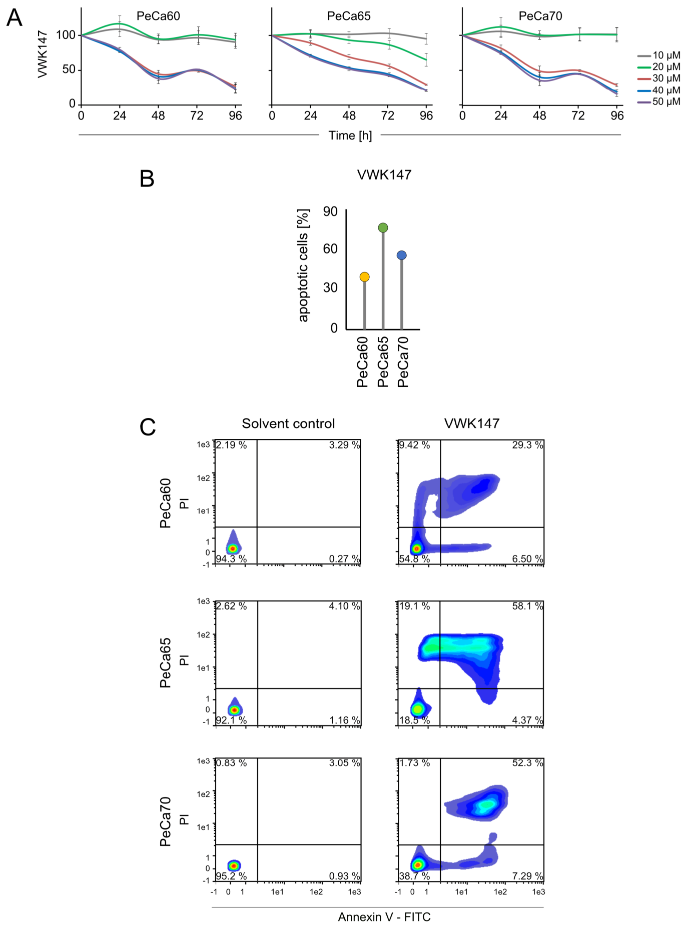

Figure S6:
A) XTT cell viability assays of PeCa60, PeCa65, and PeCa70 cells treated with VWK147 for 24 - 96 h (n = 4). B) Apoptosis analysis of PeCa60, PeCa65, and PeCa70 cells treated with VWK147 ($LD_{50\ 48\ h}$) after 48 h. C) Scatter plots showing Annexin V-FITC and propidium iodide staining with subsequent flow cytometry in PeCa cells (PeCa60, PeCa65, PeCa70) treated with the corresponding $LD_{50\ 48\ h}$ concentrations of VWK147 for 48 h. Corresponding to the data shown in Fig. S6 B.

Table S1 A: Evaluated cell lines including appropriate culture conditions.

Cell line	Species	Culture conditions	Cancer Type	Entity	Medium	Supplements	Kindly provided by
2102EP	Human	5 % CO2, 37 °C	germ cell tumor	Embryonal carcinoma	DMEM (1x) + GlutaMAX-I	10% FBS, 1% P/S (10,000 U), 1% L-Glutamin (200 mM)	Dr. Christoph Oing, Department of Oncology, Hematology and Bone Marrow Transplantation with Section of Pneumology, Mildred Scheel Cancer Career Center HaTriCs4, University Cancer Center Hamburg, Hamburg, Germany
GCT72	Human	5 % CO2, 37 °C		Yolk-sac tumor	RPMI Medium 1640 (1x)	10% FBS, 1% P/S (10,000 U), 1% L-Glutamin (200 mM)	Dr. Thomas Müller (Medical Faculty of Martin Luther University Halle-Wittenberg, Halle (Saale), Germany)
JAR	Human	5 % CO2, 37 °C		Choriocarcinoma	DMEM (1x) + GlutaMAX-I	10% FBS, 1% P/S (10,000 U), 1% L-Glutamin (200 mM)	ATCC, #HTB-144
TCam-2	Human	5 % CO2, 37 °C		Seminoma	RPMI Medium 1640 (1x)	10% FBS, 1% P/S (10,000 U), 1% L-Glutamin (200 mM)	Dr Janet Shipley, Division of Molecular Pathology, The Institute of Cancer Research, London, United Kingdom
PeCa53	Human	7.5 % CO2, 37 °C	penile cancer	penile squamous cell carcinoma	Keratinocyte Growth Medium 2	1% P/S (10,000 U), SupplementMix Keratinocyte Growth Medium 2 w/o Calcium, $CaCl_2$ - Solution	PD Dr. Johannes Linxweiler, Department of Urology and Pediatric Urology, Saarland University, Homburg, Germany
PeCa60	Human	7.5 % CO2, 37 °C		penile squamous cell carcinoma	Keratinocyte Growth Medium 2	1% P/S (10,000 U), SupplementMix Keratinocyte Growth Medium 2 w/o Calcium, $CaCl_2$ - Solution	PD Dr. Johannes Linxweiler, Department of Urology and Pediatric Urology, Saarland University, Homburg, Germany
PeCa60Xen	Human	7.5 % CO2, 37 °C		penile squamous cell carcinoma	AmnioMAX-C100 (1x) Basal Medium	AmnioMAX-C100 Supplement	PD Dr. Johannes Linxweiler, Department of Urology and Pediatric Urology, Saarland University, Homburg, Germany
PeCa60Xen^{met}	Human	7.5 % CO2, 37 °C		penile squamous cell carcinoma	AmnioMAX-C100 (1x) Basal Medium	AmnioMAX-C100 Supplement	PD Dr. Johannes Linxweiler, Department of Urology and Pediatric Urology, Saarland University, Homburg, Germany
PeCa65	Human	7.5 % CO2, 37 °C		penile squamous cell carcinoma	Keratinocyte Growth Medium 2 + AminoMAX-C100 (1x) Basal Medium (1:1)	SupplementMix Keratinocyte Growth Medium 2 w/o Calcium, $CaCl_2$ - Solution, AmnioMAX-C100 Supplement	PD Dr. Johannes Linxweiler, Department of Urology and Pediatric Urology, Saarland University, Homburg, Germany
PeCa70	Human	7.5 % CO2, 37 °C		penile squamous cell carcinoma	Keratinocyte Growth Medium 2	1% P/S (10,000 U), SupplementMix Keratinocyte Growth Medium 2 w/o Calcium, $CaCl_2$ - Solution	PD Dr. Johannes Linxweiler, Department of Urology and Pediatric Urology, Saarland University, Homburg, Germany
MPAF	Human	7.5 % CO2, 37 °C	non-cancerous controls	fibroblast	DMEM (1x) + GlutaMAX-I	10% FBS, 1% P/S (10,000 U), 1% L-Glutamin (200 mM), 1% non-ess. aa (100x), 100 nM β-Mercaptoethanole	Dr. Michael Peitz, Institute of Reconstructive Neurobiology, University of Bonn Medical Faculty and University Hospital Bonn, Bonn, Germany
LB-C18m	Human	7.5 % CO2, 37 °C		fibroblast	DMEM (1x) + GlutaMAX-I	10% FBS, 1% P/S (10,000 U), 1% L-Glutamin (200 mM), 1% non-ess. aa (100x), 100 nM β-Mercaptoethanole	Dr. Michael Peitz, Institute of Reconstructive Neurobiology, University of Bonn Medical Faculty and University Hospital Bonn, Bonn, Germany

Table S1 B: Evaluated chemotherapeutic drugs and inhibitors used in this study.

Drug class	Drug	Supplier	Order no.	Solvent
Heat shock protein 90 inhibitor	17-AAG	Selleckchem	S1141	DMSO
Antimetabolite	5-Fluorouracil	MedChemExpress	HY-90006	DMSO
Histone methyltransferase inhibitor	Chaetocin	Selleckchem	S8068	DMSO
Alkylating agent	Cisplatin	NeoCorp / Hexal	German Pharmacy Central Number (PZN): 2139417	NaCl
Taxane	Docetaxel	MedChemExpress	HY-B0011	DMSO
Alkylating agent	Ifosfamide	MedChemExpress	HY-17419	DMSO
Topoisomerase inhibitor	Irinotecan	MedChemExpress	HY-16562	DMSO
Histone demethylase inhibitor	JIB-04	Selleckchem	S7281	DMSO
Taxane	Paclitaxel	MedChemExpress	HY-B0015	DMSO
CDK4/6 inhibitor	Palbociclib	Pfizer Ltd.	MTA to D.N.: WI238366	H_2O, pH3
Polycpolycomb-repressive complex 1 inhibitor	PRT4165	Selleckchem	S5315	DMSO
Heat shock protein 90 inhibitor	PU-H71	Selleckchem	S8039	DMSO
Histone deacetylase inhibitor	Quisinostat	Selleckchem	S1096	DMSO
CDK4/6 inhibitor	Ribociclib	Novartis Pharma AG	MTA to D.N.: PLSMTA18FEB46	DMSO
Histone deacetylase inhibitor	Romidepsin	Selleckchem	S3020	DMSO
Heat shock protein 90 inhibitor	VWK147	Prof. Dr. Thomas Kurz, Dept. of Pharmaceutical and Medical Chemistry, Heinrich Heine University Düsseldorf, Düsseldorf, Germany	n.d.	DMSO

Table S1 C: Oligonucleotide sequences utilized in this study.

	Gene	Forward primer	Reverse primer	Tm	Cycles
qRT-PCR	*ACTB*	AAAGACCTGTACGCCAACAC	GTCATACTCCTGCTTGCTGAT	60°C	45
	GAPDH	TGCCAAATATGATGACATCAAGAA	GAGTGGGTGTCGCTGTTG	60°C	45
	ARID1A	TCTTGCCCATCTGATCCATT	CCAACAAAGGAGCCACCAC	60°C	45
	APAF1	ACAATGCTCTACTACATGAA GGATATAAAGA	CACTGGAAGAAGAGACAACAGGAA	60°C	45
	ATF3	AAGAACGAGAAGCAGCATTTGAT	TTCTGAGCCCGGACAATACAC	60°C	45
	AURKA	TGGGTGGTCAGTACATGCTC	TGCATCCGACCTTCAATCATTTC	60°C	45
	AURKB	CGCAGAGAGATCGAAATCCAG	AGATCCTCCTCCGGTCATAAAA	60°C	45
	AURKB	CAGAAGAGCTGCACATTTGACG	CCTTGAGCCCTAAGAGCAGATTT	60°C	45
	BAD	CCCAGAGTTTGAGCCGAGTG	CCCATCCCTTCGTCGTCCT	60°C	45
	BAK1	ATGGTCACCTTACCTCTGCAA	TCATAGCGTCGGTTGATGTCG	60°C	45
	BAX	GGGGACGAACTGGACAGTAA	CAGTTGAAGTTGCCGTCAGA	60°C	45
	BCL2	CCTGTGGATGACTGAGTACCTG	CAGAGGCCGCATGCTGGG	60°C	45
	BCL2L1	ACATCCCAGCTCCACATCAC	AAGAGTGAGCCCAGCAGAAC	60°C	45
	BIRC5	AGGACCACCGCATCTCTACAT	AAGTCTGGCTCGTTCTCAGTG	60°C	45
	BRCA1	GAAACCGTGCCAAAAGACTTC	CCAAGGTTAGAGAGTTGGACAC	60°C	45
	BRCA2	CACCCACCCTTAGTTCTACTGT	CCAATGTGGTCTTTGCAGCTAT	60°C	45
	BRD4	AGCAGCAACAGCAATGCTGAG	GCTTGCACTTGTCCTCTTCC	60°C	45
	BRD7	AAGCACAAGTCGGACAAACAC	CGTTCCCTCCTACTTTGAGGAC	60°C	45
	CASP7	AGTGACAGGTATGGGCGTTC	CGGCATTTGTATGGTCCTCTT	60°C	45
	CASP8	TTTCTGCCTACAGGGTCATGC	GCTGCTTCTCTCTTTGCTGAA	60°C	45
	CD24	AAACAACAACTGGAACTTCAAGTAACTC	GGTGGTGGCATTAGTTGGATTT	60°C	45
	CD274	GGACAAGCAGTGACCATCAAG	CCCAGAATTACCAAGTGAGTCCT	60°C	45
	CDK4	ATGGCTACCTCTCGATATGAGC	CATTGGGGACTCTCACACTCT	60°C	45
	CDK6	CTGAATGCTCTTGCTCCTTT	AAAGTTTTGGTGGTCCTTGA	60°C	45
	CDKN1A	TGGAGACTCTCAGGGTCGAAA	GGCGTTTGGAGTGGTAGAAATC	60°C	45
	CLDN6	TGTTCGGCTTGCTGGTCTAC	CGGGGATTAGCGTCAGGAC	60°C	45
	CLDN18.2	ACATGCTGGTGACTAACTTCTG	AAATGTGTACCTGGTCTGAACAG	60°C	45
	CXCR4	ACGCCACCAACAGTCAGAG	AGTCGGGAATAGTCAGCAGGA	60°C	45
	CXCR7	TCTGCATCTCTTCGACTACTCA	GTAGAGCAGGACGCTTTTGTT	60°C	45
	DHRS2	CTCCATGTAGGGCAGCAACT	GTAGGGAGCACTCTGGGGAC	60°C	45
	DUSP1	GTACATCAAGTCCATCTGAC	GGTTCTTCTAGGAGTAGACA	60°C	45
	EZH2	AATCAGAGTACATGCGACTGAGA	GCTGTATCCTTCGCTGTTTCC	60°C	45
	FADD	GCTGGCTCGTCAGCTCAAA	ACTGTTGCGTTCTCCTTCTCT	60°C	45
	FAS	AGCTTGGTCTAGAGTGAAAA	GAGGCAGAATCATGAGATAT	60°C	45
	FOS	GAGAGCTGGTAGTTAGTAGCATGTTGA	AATTCCAATAATGAACCCAATAGATTAGTTA	60°C	45
	GADD45B	GTCGGCCAAGTTGATGAAT	CACGATGTTGATGTCGTTGT	60°C	45
	HDAC1	TAAATTCTTGCGCTCCATCC	AACAGGCCATCGAATACTGG	60°C	45
	HDAC6	GAGGGAGAACTCCGTGTCCTA	AATAGCCATCCATAAGACTGTGC	60°C	45
	HSP90AA1	CATAACGATGATGAGCAGTACGC	GACCCATAGGTTCACCTGTGT	60°C	45
	HSP90AB1	CGAAGTTGGACAGTGGTAAAGAG	TGCCCAATCATGGAGATGTCT	60°C	45
	HSPA1A	GCCGAGAAGGACGAGTTTGA	TCCGCTGATGATGGGGTTAC	60°C	45
	HSPA1B	GCGAGGCGGACAAGAAGAA	GATGGGGTTACACACCTGCT	60°C	45
	ID2	TCAGCCTGCATCACCAGAGA	CTGCAAGGACAGGATGCTGATA	60°C	45
	LYN	GCTTTTGGCACCAGGAAATAGC	TCATGTCGCTGATACAGGGAA	60°C	45
	MMP2	TACAGGATCATTGGCTACACACC	GGTCACATCGCTCCAGACT	60°C	45
	MMP9	AGACCTGGGCAGATTCCAAAC	CGGCAAGTCTTCCGAGTAGT	60°C	45
	MYC	CAGCTGCTTAGACGCTGGATT	GTAGAAATACGGCTGCACCGA	60°C	45
	NECTIN4	GGACCAAAGGATCACCCACAT	CAGGCACTTGAGCATAGCTCC	60°C	45
	NOXA / PMAIP1	ACCAAGCCGGATTTGCGATT	ACTTGCACTTGTTCCTCGTGG	60°C	45
	POLE	TTGCGACCAGAAAGGGTTGT	TGATTTGGCAAGTCCAGATCCT	60°C	45
	RHOB	GGGACAGAAGTGCTTCACCT	CGACGTCATTCTCATGTGCT	60°C	45
	SLFN5	GAGTGTGTTGTAGATGCAGGAA	ACTGCTCGCAGGATGATTTCA	60°C	45
	SMARCA2	TCCGAGGCAAAATCAGTCAAG	TTCCTCGATTTGGCCTTTTCT	60°C	45
	SMARCA4	CAGCATGCCAAGGATTTCAAG	CGATCCGCTCGTTCTCTTTC	60°C	45
	SPC25	AGTACGGACACCTCCTGTCAG	TCTCAACCATTCGTTCTTCTTCC	60°C	45
	STAT3	ATCACGCCTTCTACAGACTGC	CATCCTGGAGATTCTCTACCACT	60°C	45
	TP53	CAGCACATGACGGAGGTTGT	TCATCCAAATACTCCACACGC	60°C	45
	TROP2	ACAACGATGGCCTCTACGAC	GTCCAGGTCTGAGTGGTTGAA	60°C	45
	UHRF1	GCCATACCCTCTTCGACTACG	GCCCCAATTCCGTCTCATCC	60°C	45
	VEGFA	AGGGCAGAATCATCACGAAGT	AGGGTCTCGATTGGATGGCA	60°C	45
genomic PCR	mycoplasma test primer mix	CGCCTGAGTAGTACGTTCGC	GCGGTGTGTACAAGACCCGA	65°C	34
		CGCCTGAGTAGTACGTACGC	GCGGTGTGTACAAAACCCGA		
		TGCCTGGGTAGTACATTCGC	GCGGTGTGTACAAACCCCGA		
		TGCCTGAGTAGTACATTCGC			
		CGCCTGAGTAGTATGCTCGC			
		CACCTGAGTAGTATGCTCGC			
		CGCCTGGGTAGTACATTCGC			

Table S1 D: Antibodies utilized in this study.

Antibodies	Company	Clone	Order no.	Dilution	Application
cleaved PARP	Cell Signaling Technology	Asp214	9544	1:1000	WB
HSP70	Santa Cruz Biotechnology	C92F3A-5	sc-66048	1:1000	WB
HSP90α	Santa Cruz Biotechnology	F-2	sc-515081	1:1000	WB
PARP	Cell Signaling Technology	46D11	9532	1:1000	WB
GAPDH	Abcam	6C5	ab8245	1:30000	WB
Rabbit-Anti-Mouse Immunoglobulins/HRP	Dako		P0260	1:2000	WB
Goat-Anti-Rabbit Immunoglobulins/HRP	Dako		P0448	1:1000	WB

Data S1: Overview of the most abundant proteins of the proteome (A) and secretome (B) in PeCa53, PeCa60, PeCa65, and PeCa70, and PeCa60, PeCa60Xen, and PeCa60Xen^{met} (intensity > 50,000,000). C) List of the strongest phosphorylated proteins of the phosphokinome D) List of PeCa exclusive factors identified in the proteome and secretome, which were not detected in fibroblasts (MPAF, LB-C18m, iLB-C1-30m, LB-C35m, and LB-C2-36m).

Raw data file 1: Uncropped raw western blot data. Uncropped western blot data corresponding to A) Fig S2 A and B) Fig. S2 E.

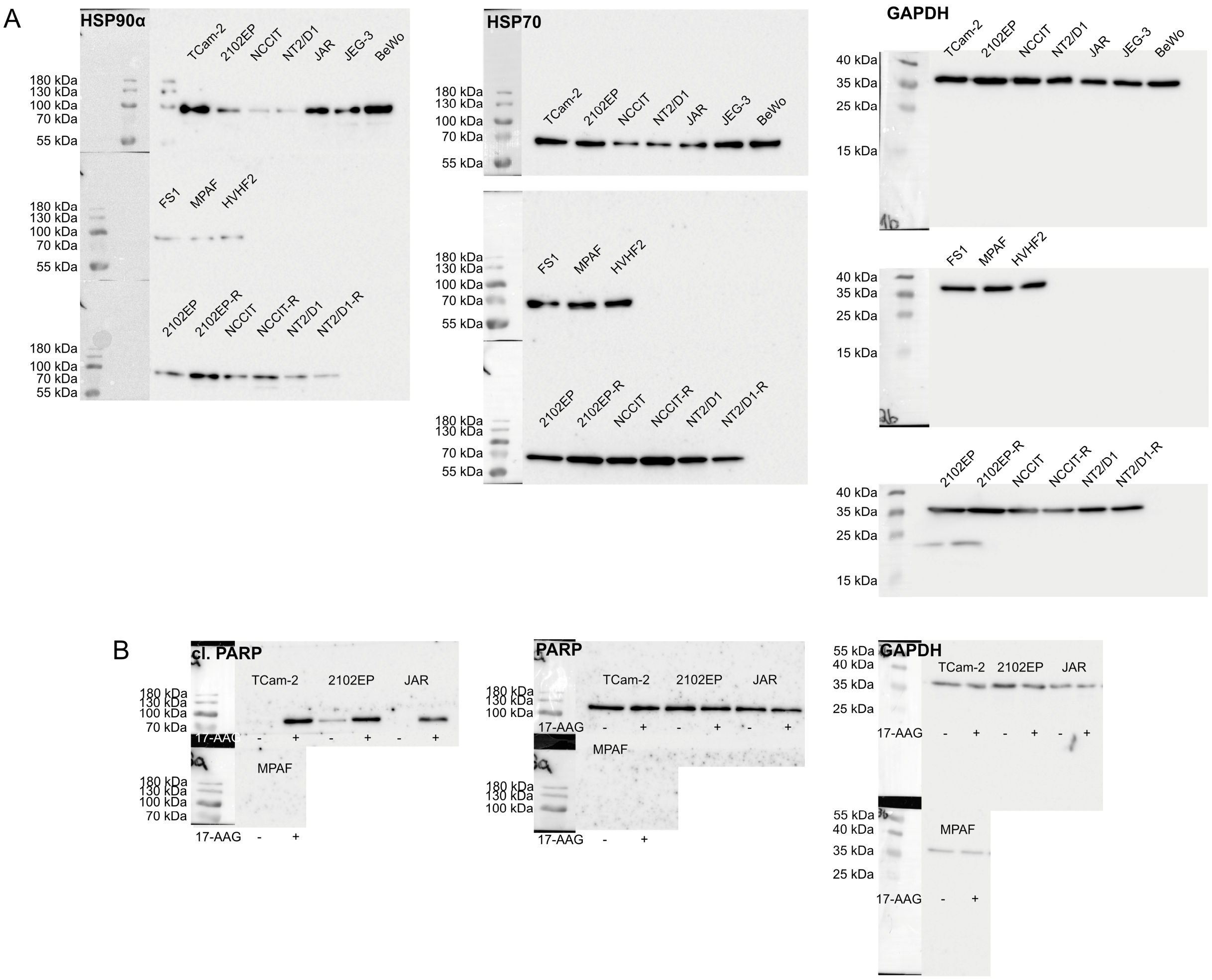

Supplement: Supplementary file 1 — Supplementary information [file 41698_2026_1391_MOESM1_ESM.pdf]
